# Development and specification of GABAergic cortical interneurons

**DOI:** 10.1186/2045-3701-3-19

**Published:** 2013-04-23

**Authors:** Corey Kelsom, Wange Lu

**Affiliations:** 1 Eli and Edythe Broad Center for Regenerative Medicine and Stem Cell Research, Department of Biochemistry and Molecular Biology, University of Southern California, 1425 San Pablo Street, Los Angeles, CA 90033, USA

**Keywords:** GABA, Interneuron, Cortex, Specification, Transcriptional network

## Abstract

GABAergic interneurons are inhibitory neurons of the nervous system that play a vital role in neural circuitry and activity. They are so named due to their release of the neurotransmitter gamma-aminobutyric acid (GABA), and occupy different areas of the brain. This review will focus primarily on GABAergic interneurons of the mammalian cerebral cortex from a developmental standpoint. There is a diverse amount of cortical interneuronal subtypes that may be categorized by a number of characteristics; this review will classify them largely by the protein markers they express. The developmental origins of GABAergic interneurons will be discussed, as well as factors that influence the complex migration routes that these interneurons must take in order to ultimately localize in the cerebral cortex where they will integrate with the neural circuitry set in place. This review will also place an emphasis on the transcriptional network of genes that play a role in the specification and maintenance of GABAergic interneuron fate. Gaining an understanding of the different aspects of cortical interneuron development and specification, especially in humans, has many useful clinical applications that may serve to treat various neurological disorders linked to alterations in interneuron populations.

## Introduction

Interneurons play a vital role in the wiring and circuitry of the developing nervous system of all organisms, both invertebrates and vertebrates alike. Generally speaking, an interneuron is a specialized type of neuron whose primary role is to form a connection between other types of neurons. They are neither motor neurons nor sensory neurons, and also differ from projection neurons in that projection neurons send their signals to more distant locations such as the brain or the spinal cord. Of great importance is that interneurons function to modulate neural circuitry and circuit activity [1-4]. A large majority of interneurons of the central nervous system are of the inhibitory type. In contrast to excitatory neurons, inhibitory cortical interneurons characteristically release the neurotransmitters gamma-aminobutyric acid (GABA) and glycine [5-7]. Cortical interneurons are so named for their localization in the cerebral cortex, which is defined as a sheet of outer neural tissue, that functions to cover the cerebrum and cerebellum structures in the brain.

This review will place an emphasis on the function and origin of GABAergic cortical interneurons of the developing nervous system. Within the overarching categorization of GABAergic interneurons there are also numerous interneuron subtypes that are largely categorized based on the surface markers they express. Three major cortical interneuron subtypes will be discussed: parvalbumin (PV)-expressing interneurons, a heterogeneous population of somatostatin (SST)-expressing interneurons, and a relatively recently identified population of the ionotropic serotonin receptor 5HT3a (5HT3aR)-expressing interneurons; together, these three subtypes account for approximately 100 percent of the mouse neocortical GABAergic interneuronal population [8]. Although these interneurons home to their respective layers of the cerebral cortex, they are generated in various subpallial locations; the primary origins of these interneuronal progenitors will be extensively discussed as well. The subsequent migratory routes that these interneuronal precursors take to the cerebral cortex are also covered here.

Of particular interest as of late in this field of research is mapping the network of transcription factors that are responsible for specifying cortical interneurons and specific interneuronal subtype. This review is organized such that the transcription factors are organized and discussed based on the major interneuronal origins in the subpallium. The most arduous task that researchers face is understanding the mechanism by which *each* interneuron subtype is specified as well as the various genes and transcription factors that may be involved; this is not helped by the fact that there are so many subtypes whose features often overlap with one another.

Of utmost importance with regard to cortical circuitry is the balance between excitatory inputs and inhibitory inputs that must be carefully maintained. Disrupting the balance of neural circuits may very well be a contributing factor toward the emergence of neuropsychiatric disorders, such as epilepsy, autism spectrum disorders, and intellectual disabilities, just to name a few [9]. This review focuses on one severe mental illness in particular, schizophrenia. Gaining an understanding of cortical interneurons within the “bigger picture” of neocortical circuitry may provide much needed information behind the etiologies of neurological disorders.

### Role of GABAergic cortical interneurons

Given that the population of GABAergic interneurons in the brain is such a heterogeneous one, it is only logical that the many different classes of interneurons will have a myriad of roles to play in the adult nervous system. GABAergic neurons play an inhibitory role and synaptically release the neurotransmitter GABA in order to regulate the firing rate of target neurons. Neurotransmitter release typically acts through postsynaptic GABA_A_ ionotropic receptors in order to trigger a neuronal signaling pathway.

This research field typically organizes interneuron role/function into three components: (1) afferent input, (2) intrinsic properties of the interneuron, and (3) targets of the interneuron. Generally speaking, interneurons receive input from various sources, including pyramidal cells as well as cells from other cortical and subcortical regions [10,11]. With regard to output, cortical interneurons engage in feed-forward and feedback inhibition [12-14]. Regardless of the mode of output, the cortical interneuronal network is further complicated by the fact that a single cortical interneuron is capable of making multiple connections with its excitatory neuronal target(s) [15].

### Cortical interneuron subtype

It is estimated that there are over 20 different subtypes of GABAergic interneurons in the cortex, and subtypes are also distinguished from one another based upon the calcium-binding proteins they express, which serve as markers (Table 1) [16-24]. Studies performed in both mouse and rat brain tissue have suggested that in particular, the calcium-binding protein known as parvalbumin, and the neuropeptide somatostatin, are two crucial markers in defining the most predominant interneuron subtypes within the cerebral cortex [16,20,22]. Importantly, the PV-expressing interneuron population is independent from the SST-expressing population, in that expression of these markers does not overlap [16,20,22,25]. In addition to PV- and SST-positive GABAergic interneurons, which together comprise approximately 70% of the total GABAergic cortical interneuron population, another subgroup of interneurons that express 5HT3aR were found to comprise approximately 30% of all interneurons [25]. While these three interneuronal subpopulations account for nearly (if not all) 100% of all GABAergic cortical interneurons, it is also important to remember that each of these populations, especially the 5HT3aR-expressing population, is heterogeneous, and therefore expresses other proteins or neuropeptides that contribute to their characterization.

**Table 1:** GABAergic cortical interneuron subtypes

**Marker**	**% Total GABA + Population**	**Morphology**	**Axonal targeting**	**Firing pattern**	**Origin**
Parvalbumin (PV)	40	Basket cells	Proximal dendrites/soma	Fast-spiking	Ventral MGE
		Chandelier cells	Axonal initial segment		
Somatostatin (SST)	30	Martinotti cells	Distal dendrites	Bursting	Dorsal MGE
5HT3aR	30	VIP+: Small bipolar	Proximal dendrites	Irregular-spiking,	CGE
		VIP-: Neurogliaform cells	Other GABA neurons	Fast-adapting	
				Late spiking accommodating	

Three markers account for nearly 100 percent of the GABAergic cortical interneuron population: parvalbumin (PV), somatostatin (SST), and 5HT3a receptor (5HT3aR). Outlined in this table are percentage of total GABA-expressing neurons, morphology, axonal targeting, firing pattern, and origin based on the type of marker expressed.

In the recent years there has been a push to create a consistent nomenclature for the varying interneuronal subtypes; a 2005 conference in Petilla, Spain, was held to accomplish this task. A group of researchers known as the Petilla Interneuron Nomenclature Group (PING) convened to formulate a set of terminologies to describe the morphological, molecular, and physiological features of GABAergic cortical interneurons [16]. Morphologically speaking, cortical interneurons are described with regard to their soma, dendrites, axons, and the connections they make. Molecular features include transcription factors, neuropeptides, calcium-binding proteins, and receptors these interneurons express, among many others. Physiological characteristics include firing pattern, action potential measurements, passive or subthreshold parameters, and postsynaptic responses, to name a few [16]. The overarching goal of this conference and the resulting Petilla terminology is to create a uniform set of criteria by which interneurons can be described so as to reduce confusion between the findings by various research groups in this field. While results from the Petilla conference have shown that there are many criteria with which to define and distinguish classes of interneurons, this review will classify interneuron subtypes based on the markers they express, particularly PV, SST, or 5HT3aR, and elaborate upon each of these three groups.

#### Parvalbumin-expressing interneurons

The PV interneuron group represents approximately 40% of the GABAergic cortical interneuron population [8]. This population of interneurons possesses a fast-spiking pattern, and fire sustained high-frequency trains of brief action potentials [10,16,17,22,26]. Additionally, these interneurons possess the lowest input resistance and the fastest membrane time constant of all interneurons [10,16,17,22,23,27,28].

Two types of PV-interneurons make up the PV interneuron group: basket cells and chandelier cells [16,22]. Far more is understood about basket cells, which are interneurons that make synapses at the soma and proximal dendrite of target neurons, and usually have multipolar morphology [16,22]. Several studies have shown that fast-spiking basket neurons are the dominant inhibitory system in the neocortex, where they mediate the fast inhibition of target neurons, among many other functions [29-35]. As will be discussed in a later section, fast-spiking basket neurons likely play a large role in regulating the delicate balance between excitatory and inhibitory inputs in the cerebral cortex [36,37].

Much less is known about the second subgroup of PV-expressing interneurons, the chandelier cells. Unlike basket neurons, chandelier cells target the axon initial segment of pyramidal neurons [16,22]. Both basket cells and chandelier cells are fast-spiking, but they differ in electrophysiological properties, as reviewed by Woodruff *et al*[38]. Several relatively recent studies have suggested that in contrast to other interneurons, chandelier cells may be excitatory rather than inhibitory due to their depolarizing effects on membrane potential [38-40], although the functions of this subgroup have yet to be elucidated.

One research group has characterized a group of PV-expressing cells that is independent from chandelier and basket neurons in the mouse neocortex [41]. These interneurons were designated multipolar bursting cells, and differ from chandelier and basket cells in both electrophysiology and connectivity [41]. Multipolar bursting neurons possess synapses with pyramidal cells (or other multipolar bursting cells) that demonstrate a paired-pulse facilitation; in contrast, chandelier and basket cells are usually strongly depressing [41]. While this group of interneurons holds promise as a third subgroup within the PV-expressing interneuron type, further investigation is warranted, as no other research groups have characterized these neurons.

#### Somatostatin-expressing interneurons

The SST-expressing interneuron group is the second-largest interneuron group in the mouse neocortex, representing roughly 30% of the total cortical interneuron population [8]. SST-positive interneurons are known as Martinotti cells, and possess ascending axons that arborize layer I and establish synapses onto the dendritic tufts of pyramidal neurons [22]. Martinotti cells are found throughout cortical layers II-VI, but are most abundant in layer V [14,22,42]. These interneurons function by exhibiting a regular adapting firing pattern but also may initially fire bursts of two or more spikes on slow depolarizing humps when depolarized from hyperpolarized potentials. In contrast to PV-positive interneurons, excitatory inputs onto Martinotti cells are strongly facilitating [43-47]. More details regarding the electrophysiology and firing patterns of SST-expressing Martinotti cells can be found in a review by Rudy *et al*[8].

Results from several studies have suggested that the SST interneuron population is a heterogeneous one. Ma *et al* generated a mouse line designated as X94, whereby GFP expression (encoded for by random insertion of the GFP gene) was controlled by the GAD67 promoter; cells from this line express SST but differ from Martinotti cells in many aspects [48]. These cells were located in layers IV and V that, unlike, Martinotti cells, targeted cells from cortical layer IV [48]. X94 cells also possessed a lower input resistance relative to Martinotti cells, with spikes of a shorter duration and a stuttering firing pattern [48]. This evidence clearly suggests that there are other interneuronal subgroups within the SST subtype than just Martinotti cells. McGarry and colleagues have reported that another transgenic mouse line contains two distinct, SST-positive cells that primarily occupy layers II and III [49]. Like the X94 cells, these two populations of interneurons differ electrophysiologically than Martinotti cells, although additional research must be performed to truly determine their presence, as other research groups have not been able to duplicate these findings [26,50,51].

It is clear that there are likely additional subpopulations of SST-expressing cortical interneurons. This is bolstered by the observed differences in firing properties, expression of molecular markers, and connectivity of different neurons within this population [8,49,50,52,53]. Additional research in this field is warranted, as the SST-expressing population of GABAergic interneurons is such a large one.

#### 5HT3aR Interneuron group

The third group of GABAergic cortical interneurons was initially characterized in a study by Lee *et al*, and is designated as the 5HT3aR interneuron group [25]. This study utilized both *in situ* hybridization and immunohistochemistry to demonstrate the existence of a population of GABAergic interneurons in the mouse cortex that express the 5HTa3 receptor, but neither PV nor SST; this population accounts for approximately 30% of the GABAergic cortical interneuron population [25]. Due to its relatively recent discovery, this group has yet to be fully characterized, although it is evident that this population is a very heterogeneous one.

Within the 5HT3aR interneuron group are several subsets of interneurons that also express other protein or neuropeptide markers, one of them being vasoactive intestinal peptide (VIP) [22,26,54]. VIP-expressing interneurons are localized in cortical layers II and III, and while they express neither PV nor SST, Lee *et al* confirmed that this subset does indeed express the 5HTa3 receptor and accounts for approximately 40% of the 5HT3aR population [25]. VIP interneurons generally make synapses onto dendrites [25,55,56], and some have been observed to target other interneurons [57,58]. Relative to all cortical interneurons, VIP interneurons possess a very high input resistance and are among the most excitable of interneurons [25,55,56].

There are several types of VIP cortical interneurons that differ in electrophysiological properties, but in general they possess a bipolar, bitufted and multipolar morphology [22,26,54,56]. One VIP subtype in particular is commonly referred to as irregular-spiking neurons [17,25,55,56,59-61]. Irregular spiking interneurons possess a vertically oriented, descending axon that extends to deeper cortical layers, and have an irregular firing pattern that is characterized by action potentials occurring irregularly during depolarizations near threshold [17,25,55,56,59-61]. Additionally, irregular spiking cortical interneurons express the calcium-binding protein calretinin (CR) [56,61], which is a marker that some SST-positive interneurons also possess (and interestingly, also a marker that neither VIP nor SST interneurons express) [25,55,56,62].

There are several other subtypes of VIP-expressing 5HT3aR interneurons present in the cerebral cortex, which are nicely summarized by Rudy *et al*[8]. Briefly, among these are rapid-adapting [63], fast-adapting neurons [25,56] and IS2 [61], as well as a minor population of VIP-positive basket cells with regular, bursting, or irregular-spiking firing patterns [22,26,64].

60% of cortical interneurons in the 5HT3aR-expressing group do not express VIP [25]. Of this VIP-negative 5HT3aR group, nearly 80% express the interneuronal marker reelin [56]. Neurogliaform cells are a type of cortical interneuron that belongs to this category: they are also known as spiderweb cells and express neuropeptide Y (NPY), with multiple dendrites radiating from a round soma [22,65]. Neurogliaform cells are unique relative to other GABAergic cortical interneurons because they are capable of forming synaptic connections with each other *as well as* with other interneuronal types (as opposed to other interneurons that can only make synapses onto homologous neurons), thus solidifying their important role in regulating neural circuitry [66-68]. Furthermore, neurogliaform cells function by activating slow GABA_A_ and GABA_B_ receptors in order to provoke long lasting inhibitory postsynaptic potentials onto pyramidal neurons and other interneurons [65,69]. Other subtypes exist within the 5HT3aR-positive, VIP-negative group as well; see the 2010 review by Rudy *et al*[8]. While progress has certainly been made in distinguishing interneuron groups from one another, further research is definitely warranted.

### Origin of GABAergic cortical interneurons

#### Cortical interneurons are born in the ganglionic eminences

**Figure 1:**
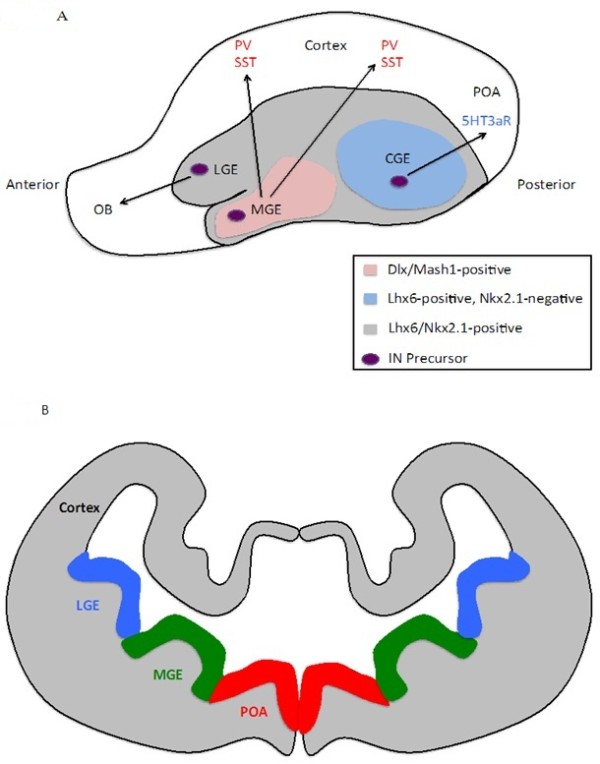
**Origin of GABAergic Cortical Interneurons.** Anatomy of the embryonic telencephalon at approximately embryonic day 13.5 (E13.5), showing the major origins of GABAergic cortical interneurons. **A** Sagittal (top) view of the telencephalon. The MGE is labeled in pink and represents Lhx6- Nkx2.1-positive areas. MGE-derived interneurons ultimately express either PV or SST in the cerebral cortex. The CGE is labeled in light blue and represents Lhx6-positive, Nkx2.1-negative areas. CGE-derived interneurons ultimately express 5HT3aR in the cortex. The gray-labeled area represents Dlx1/Mash1-expressing areas. The black dotted line represents the migratory route interneuron precursors take to the cortex. **B** Coronal view of the telencephalon. Interneuronal progenitors originating in the LGE are labeled in blue; MGE-derived progenitors are labeled in green, and interneurons from the preoptic area are labeled in red. Abbreviations: CR, calretinin; CGE, caudal ganglionic eminence; IN, interneuron; LGE, lateral ganglionic eminence; MGE, medial ganglionic eminence; OB, olfactory bulb; PV, parvalbumin; POA, preoptic area; SST, somatostatin.

Throughout embryogenesis, interneurons are primarily generated in a structure broadly termed the ganglionic eminence (GE) (Figure 1) [70]. The GE is a transitory brain structure located in the ventral area of the telencephalon, and is anatomically present during embryonic development. The GE becomes evident at approximately E11.5 in the developing murine system [71]. In total there are three ganglionic eminences: the medial ganglionic eminence (MGE), the caudal ganglionic eminence (CGE), and the lateral ganglionic eminence (LGE). The names of the different areas within the GE are based on their rostral-caudal location in the telencephalon. As embryonic development continues, the GEs grow and ultimately fuse, at which point they are no longer present in the mature brain. The MGE and CGE are the primary sources of cortical interneurons in the developing nervous system.

Several crucial studies during the 1980s demonstrated that contrary to the previous postulation that cortical interneurons originate in the underlying cortical ventricular zone, the majority of GABAergic interneurons are actually born in the ganglionic eminences. Van Eden and colleagues first showed, utilizing analysis of GABA-positive cells in the developing rodent cerebral cortex, that cells presumed to be GABAergic interneurons seemed to be migrating *away* from the subpallium [72]. This crucial observation spawned several other studies that subsequently discovered that indeed, interneurons must migrate from the subpallium to their final destinations in the cortex. To corroborate Van Eden *et al*’s findings, BrdU labeling of cells in the subpallium were found to accumulate in the cerebral cortex throughout the course of neurogenesis; importantly, these BrdU labeled cells were GABA-positive, implicating these cells as cortical interneurons [73]. Additional studies utilizing fluorescent dye labeling of neurons and an *in vitro* migratory assay with DiI showed that these interneurons display the same migratory pattern from the ganglionic eminence to the mature cerebral cortex [74,75]. Use of retroviruses to track neural cell lineage also contributed the early yet important study that excitatory and inhibitory neurons are actually generated independently from one another and do not share a common lineage [76,77]. Rakic and Lombroso later confirmed that the telencephalon consists of two separate domains: one containing the GEs that serves as the source of inhibitory, GABAergic interneurons, and a second in which excitatory neurons are generated [78]. The formerly mentioned domain is also termed the subpallial compartment, whereas the latter is referred to as the pallial compartment. Later studies demonstrated that distinct cortical interneuron subtypes are derived from specific regions within the GEs, as will now be discussed.

#### Medial ganglionic eminence

The medial ganglionic eminence has long been regarded as a primary source of GABAergic cortical interneurons; it is thought to be the site of origin of approximately~ 50-60% of the cortical interneuron population in mice [63,79,80]. With regard to interneuron subtype, the MGE gives rise to most of the PV- and SST-expressing interneuron population [80]. To break this down, studies have demonstrated that *in vitro*, approximately 65% of MGE-derived interneurons express parvalbumin, with the remainder of the MGE-derived interneuronal population (~35%) expressing somatostatin [81,82]. *In vivo* experiments have confirmed these initial findings [63,81,83-85]. Of note, the SST-positive subset that is derived from the MGE is a heterogenous population with regard to morphology, electrophysiology, and expression of reelin, NPY, and/or CR [50,56].

It remains to be elucidated how PV-expressing and SST-expressing GABAergic cortical interneurons are spatially segregated within the MGE itself, and to what extent this spatial segregation occurs. Flames *et al* have suggested that the MGE may actually consist of several progenitor domains, each of which may serve as an origin for unique classes of interneurons [84].

#### Caudal ganglionic eminence

The CGE itself is unique in that it is somewhat of a “hybrid” of the MGE and LGE: similar to the MGE, the ventral-most CGE expresses transcription factor Nk×2.1, and the dorsal region of the CGE expresses the transcription factor Gsh2, which is required for proper patterning of the LGE [86]. The caudal ganglionic eminence has been shown to be the second-greatest contributor of interneuron progenitors, producing approximately 30-40% of all cortical interneurons [87-89]. While this is a substantial percentage, the CGE proves to be a difficult place of origin to objectively study due to the challenge of consistently defining this region. Fate mapping CGE-derived interneurons and subsequently determining the exact proportion these interneurons make up within the whole cortical interneuron population has therefore proven to be quite problematic. Nery *et al* were the first to show, via *in utero* transplantation analyses, that a certain percentage of interneurons destined for the cortex indeed originates in the CGE [89]. Other studies have utilized both *in vitro* and *in vivo* experiments to confirm this initial discovery, and have further added that CGE-derived interneurons are bipolar or double-bouquet in morphology [63,79]. These interneurons express CR (not SST) and/or VIP [63,79]. Recently, Miyoshi and colleagues utilized genetic fate mapping approach to corroborate the finding that the CGE is indeed a source of cortical interneurons [56].

Findings from several studies suggest that 5HT3aR-expressing cortical interneurons are actually largely CGE-derived, as demonstrated by EGFP visualization in the *5HT3aR*-*BAC*^*EGFP*^ mouse [90-92]. Lee *et al* combined the Nkx2.1-BAC^cre^ and the cre-dependent red R26R^tdRFP^ reporter with *5HT3aR*-*BAC*^*EGFP*^ in order to exclude the MGE as a possible source of this population of interneurons [25]. Overlap of cells labeled in red and cells labeled in green was not observed, and since the Nk×2.1-BAC^cre^ line labels MGE-derived cortical interneurons, the MGE can thus be discounted as a source of 5HT3aR interneurons [25]. Additionally, the Mash1-BAC^CreER^; R26R^tdRFP^ mouse line, which is CGE-specific, displayed significant and near complete overlap with 5HT3aR-expressing cells, confirming the CGE as the major source of 5HT3aR-positive cortical interneurons [25].

#### Embryonic preoptic area (POA)

A very recent study by Gelman *et al* has demonstrated that in addition to the ganglionic eminences, the embryonic preoptic area (POA) should also be considered a source of cortical GABAergic interneurons [93]. The POA is a region of the hypothalamus, and results from this study suggest that this area contributes approximately 10% of all GABAergic interneurons in the murine cerebral cortex.

#### Lateral ganglionic eminence

While it is largely agreed upon that the MGE and CGE serve as the primary source of cortical interneurons in the developing rodent nervous system, the possibility of the LGE as a third source has also been heavily debated. Results from several studies have allowed the conclusion that the LGE is at most a minor contributor of interneurons [85,87,94]. However, observations from a few studies do suggest otherwise: Sussel and colleagues reported that *Nkx2*.*1* mutants, in which normal MGE tissue fails to form, show a 50% reduction in cortical interneuron numbers relative to wild-type [95]. If the MGE was the origin of the majority of cortical interneurons, only a 50% reduction implies that there are clearly other areas of the brain responsible for generating interneurons. More convincing evidence has shown that at E15.5, the LGE-like region in *Nkx2*.*1* mutants demonstrates strong cellular migration to the developing cortex [87,88]. BrdU labeling of neural progenitors also supports the notion of a cellular migratory route from the LGE to the cortex during embryogenesis, although only a portion of the BrdU labeled cells were also GABA-positive [87]. Additionally, Jimenez and colleagues discovered that when the MGE is removed in explants taken from rat embryos, cellular migration from the LGE to the cortex continued to be observed, suggesting that the migrating cells are *not* MGE cells merely passing through the LGE [96].

### Specification of cortical interneurons

**Figure 2:**
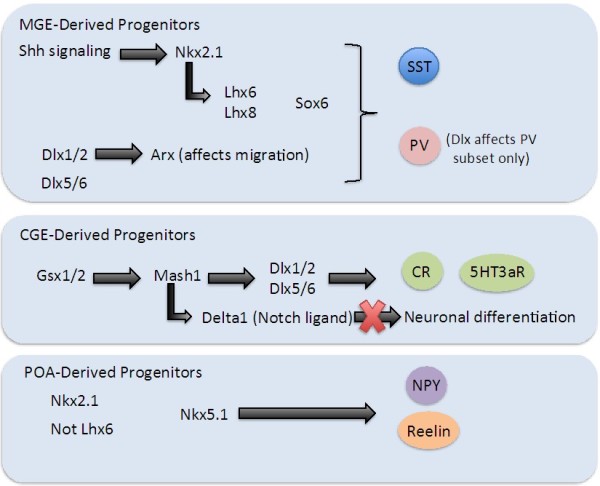
**Specification of GABAergic Cortical Interneurons.** With regard to the specification of MGE-derived interneuronal progenitors, several transcription factors play a role. Shh signaling activates Nk×2.1, which is the key transcription factor in specifying PV- and SST-positive interneurons from this region. Lh×6 and Lh×8 are transcription factors that lie downstream of Nk×2.1; they also aid in the specification of PV and SST interneurons (see text). Sox6 lies downstream of both Nkx2.1 and Lhx6/8. The Dlx homeobox family of genes play a key role in specification of CGE-derived cortical interneurons, although they also function to maintain the PV-expressing subset of MGE-derived interneurons (Dlx5 in particular). Arx is a homeobox transcription factor whose expression is directly affected by Dlx genes; Arx seems to play a role in the migration of interneurons to the cortex. Gsx1 and Gsx2 are both required for the specification of cortical interneurons that originate in the CGE. Mash1 is a downstream transcription factor whose absence results in reduced cortical interneuron numbers; it is required for proper function of the Notch ligand Delta1, which, in the Notch signaling pathway, serves to repress neuronal differentiation. The Dlx genes lie further downstream and play a crucial role in CGE-derived interneuron specification. The molecular mechanisms behind POA interneuron specification are unclear, although Nk×2.1 is expressed by interneurons derived from this area. Lh×6 is not expressed by these interneurons. Nk×5.1 was shown to affect the specification of NPY and Reelin interneurons.

There has been a recent push in the study of GABAergic interneurons to understand and create a transcriptional network that regulates GABAergic interneuron development, migration to the cortex, and ultimately maturation to the appropriate adult phenotype. To understand the complexity of the transcriptional network and identify other candidate genes involved in the production and patterning of interneurons in the mammalian cortex, it is most logical to separate the various transcription factors based on the three primary origins of cortical interneurons: the medial ganglionic eminence, the caudal ganglionic eminence, and the embryonic preoptic area (Figure 2).

#### Specification of MGE-derived GABAergic cortical interneurons

There is an extensively studied transcriptional network that plays a role in regulating proper development and specification of MGE-derived GABAergic cortical interneurons (Figure 2). While transcription factors such as the Dlx homeobox genes, Lh×6, and So×6 are crucial toward specification of the PV-positive and SST-positive subset of interneurons derived from the MGE, the major transcription factor that must be taken into account is Nk×2.1 [97-102], whose expression is localized and confined within the MGE [103,104]. Nk×2.1 expression dictates the generation of both PV- and SST-expressing MGE progenitors, and maintenance of Nk×2.1 expression is activated by Sonic hedgehog (Shh) signaling [105]. More specifically, the *level* of Shh signaling within MGE interneuronal progenitor cells seems to be a determinant of what marker these neurons will express, either PV or SST: Xu *et al* have shown that high levels of Shh signaling preferentially give rise to SST-expressing interneurons, which in turn results in reduced production of PV-expressing interneurons [106]. Complete absence or conditional loss of Nk×2.1 results in reduction of PV- and SST-expressing interneurons, demonstrating its importance for MGE-derived interneuronal specification [99,100,102].

A second transcription factor whose role is crucial to MGE-derived interneuronal specification, and whose expression is also confined to the MGE, is Lh×6 [99,100,102]. Lh×6 was discovered to be a target of Nk×2.1 [107]. Mutational analyses with regard to Lh×6 have demonstrated its importance in determining the fate of both PV- and SST-positive interneurons: in the absence of Lhx6, MGE-derived neural progenitors are still able to migrate properly to the pallium, but most of these interneurons fail to express either PV or SST; interestingly, an increase in NPY expression is observed [99,100,102]. Additionally, Lhx6-deficient interneurons are unable to properly integrate into their respective cortical layers; this observation suggests that factors downstream of Lhx6 contribute to the process of cortical integration [102]. Despite these findings, the PV- and SST-expressing population of cortical interneurons is not completely eliminated in *Lhx* mutants, implying that PV-positive and SST-positive interneurons are not solely derived from the MGE [99,100,102]. Alternatively, transcription factors other than Nkx2.1 and Lh×6 may be important for the specification of MGE-derived, PV and SST interneurons; this may partially compensate for the loss of Nk×2.1 and Lh×6. Additional studies should be performed to discern other origins of these subsets of cortical interneurons.

It is also important to take into account additional transcription factors that lie downstream of both Nk×2.1 and Lh×6 so as to obtain a better understanding of the specification of MGE-derived interneurons. Recent studies have done just that, and several transcription factors are suggested to act in conjunction with either Nk×2.1 or Lh×6. One such transcription factor is So×6. So×6 is an HMG-box-containing transcription factor that is expressed by MGE-derived GABAergic cortical interneurons; mice lacking *Sox6* do not possess PV-positive interneurons and have significant reductions of SST-positive interneurons [97,98]. Interestingly, proper Lh×6 functioning is required for maintenance of So×6 expression in neurons that are actively migrating toward the cortex, but not in MGE-derived interneuronal progenitors [98,108].

Another transcription factor that works in conjunction with Lh×6 is Lh×8, which lies downstream of Nk×2.1 and is co-expressed with Lhx6 in MGE-derived neuronal progenitors [95,109]. While Lh×8-positive cells are not specified to become GABAergic cortical interneurons, mutational analyses of mice lacking both Lh×6 and Lh×8 provide some insight toward the Lh×8’s role in cortical interneuron specification. *Lhx6*/*8* double mutants exhibit significantly reduced MGE-derived interneuron production and defective migration [108]. However, this phenotype is not observed in mice that are mutant for Lh×8 only [108].

The Dlx family of homeobox genes, specifically Dlx1, 2, 5, and 6, also play a role in the specification of MGE interneuronal progenitors, and are expressed in most subpallial neural progenitor cells [110,111]. The Dlx genes will be discussed more extensively in the following section, seeing as they play a larger role in the specification of CGE-derived cortical interneurons. Dl×5 in particular is expressed in the PV-positive mature interneuronal subset [101]. Wang *et al* utilized transplantation experiments to demonstrate that loss of Dl×5 or both Dl×5 and 6 in mice manifests as a significant reduction in PV-expressing interneuron numbers, alteration in dendritic morphology, and epilepsy [101].

#### Specification of CGE-derived GABAergic cortical interneurons

As was previously mentioned, approximately 30% of GABAergic cortical interneurons originate from the CGE. While it was previously thought that the gene expression profile of the CGE was merely an extension of that of the LGE [80,84,110,111], more recent work has suggested that the CGE possesses a unique transcriptional network of its own, making it a bona fide source of cortical interneurons, separate from any other source [111-113].

Of great importance in the specification of CGE-derived interneurons are the homeobox transcription factors known as Gs×1 and Gs×2, both of which are required for the specification of interneuronal progenitors in this region [106]. Xu *et al* have shown through conditional loss and gain of Gs×2 function that Gs×2 has a hand in the generation of CR-expressing bipolar cortical interneurons [106].

Investigation of genes downstream of Gs×1 and Gs×2 resulted in the discovery of Mash1, a basic-helix-loop-helix transcription factor also involved in the interneuron specification process. *Mash1* mutants demonstrate significant neuronal loss early in development, as well as reduced cortical interneuron numbers [114]. Mash1 was found to be required to express the Notch ligand Delta1, whose function within the Notch signaling pathway serves to repress neuronal differentiation [114-116]. Thus, loss of Mash1 results in the premature differentiation of cells within the subventricular zone (SVZ) expressing the Dlx genes [114,115].

As was mentioned in the previous section, the Dl× family of genes are key players in the specification of CGE-derived interneurons. The Dlx genes are located downstream of both Gsx2 and Mash1 [117-120]. With regard to interneuron specification, four Dl× genes – Dl×1, Dl×2, Dl×5, and Dl×6 – are expressed in the developing forebrain, and are central to the specification process [121]. Temporal expression of these four genes follows a Dl×2, Dl×1, Dl×5, and Dl×6 sequence [122,123]. Gs×2 and Mash1 neural progenitor cells have been shown to simultaneously express Dl×1/2 [110,115,124]. Loss of Dl×1/2 function results in the failure of Dl×5/6 expression [110,115,124]. Importantly, these *Dlx1*/*2* double mutants exhibit incorrect specification of the LGE/CGE, with inappropriate expression of ventral cortex markers [110,111]. A detailed analysis of the transcriptional network within the *Dlx1*/*2* double mutants has resulted in the investigation of almost 100 transcription factors that may play a role, both dependently and independently of the Dl× genes, in CGE-derived cortical interneuron specification [110,111]. An outline of several candidate transcription factors is nicely reviewed by Gelman *et al*[125].

#### Specification of embryonic POA-derived GABAergic cortical interneurons

While the embryonic POA is similar to the MGE in that it expresses the transcription factor Nk×2.1, many POA cells do not express the transcription factor Lh×6 [84]. A study by Gelman *et al* has elegantly showed via fate mapping with a Cre line expressed from transcription factor Nk×5.1 that the POA serves as a source of GABAergic interneurons expressing NPY and/or reelin [126]. Interestingly, none of the other calcium-binding proteins, such as PV, SST, CR or VIP, were expressed in these POA-derived cortical interneurons [126]. It is possible that this population of NPY- and/or reelin-expressing cells may stem from both the CGE and the POA, but additional investigations should be carried out to confirm these speculations. Studies should also be performed to discover the transcription factors and the roles they play in specification of POA-derived cells.

#### Influence of non-autonomous cell factors on GABAergic interneuron identity

Non-cell autonomous factors and the role they possess in conferring GABAergic cortical interneuron identity must also be taken into account. Lodato *et al* have shown that certain excitatory neurons are able to control the distribution of GABAergic interneurons within the cortex [127]. Deletion of subcerebral projection neurons and subsequent replacement with callosal projection neurons results in the abnormal lamination of cortical interneurons, along with abnormal GABAergic inhibition [127]. Certain factors may also affect particular subsets of interneurons. Orthodenticle homeobox 2 (Otx2) is a homeoprotein whose expression is required to both open and close a critical period of neural plasticity in the visual cortex of PV-expressing interneurons in mice [128,129]. It remains to be clarified whether other factors affect the specification of other interneuronal subsets in a non-cell-autonomous manner.

#### Differences in cortical interneuron origin and specification between rodents and primates

Studies in both primates and humans have determined that differences do exist between rodents and primates with regard to the origins and migratory routes of GABAergic interneurons. This will be briefly discussed, but for more detail refer to the review by Petanjek *et al*[130].

A few studies have shed light on the fact that the majority of primate GABAergic interneurons, in contrast to rodent cortical interneurons, may not actually originate solely in the GEs [131,132]. In fact, cortical interneurons in primates have also been shown to have their origins in the proliferative zones of the dorsal telencephalon [131,133-135]. Letinic and colleagues utilized retroviral labeling in organotypic slice cultures to demonstrate that there are two independent lineages of cortical interneurons in the fetal human forebrain [131]. However, whereas it was previously mentioned that the GE is the primary source of rodent interneurons, this study demonstrated that in a Dl×1/2-positive, Mash1-negative lineage of cortical interneurons in the fetal human forebrain, only 35% of the interneuron population in the proliferative ventricular zone and subventricular zone originates from the GE [131]. To support these findings, the same results were reported in the macaque monkey (*Macaca rhesus*, *Macaca fascicularis*) [134].

In addition to differing locations of interneuron origin between species, it is also important to realize that the transcription factors expressed by rodent interneurons and interneuronal precursors may not necessarily overlap with the transcriptional network that governs the development of human GABAergic interneurons. Research to better characterize human interneurons is underway. One such study investigated the expression of transcription factors Nk×2.1, Dlx1/2, Lh×6 and Mash1 in human fetal forebrains during the first half of gestation [136]. All transcription factors were expressed in both the GEs and the ventricular/subventricular zones, and expression was maintained up to 20 gestational weeks. Collectively the data suggest that cortical interneuron populations exist in multiple locations, both ventrally (as described in rodents) and dorsally in the VZ/SVZ [136]. The possibilities that the production of interneurons may follow a different time-course based on origin, or that different areas of production may contribute to the heterogeneity of the cortical interneuron population, should also be taken into account. Findings from a previous study utilizing cryosections and *in vitro* data also support the existence of multiple sources of cortical interneuron progenitors in the developing human brain [137]. The greater complexity characterizing progenitor populations most likely reflects the higher brain functioning characteristic of humans compared to other organisms.

### Migration to the cerebral cortex

Once generated and specified in their respective origins within the ventral telencephalon, GABAergic cortical interneurons face the task of migrating to their ultimate destinations within the cerebral cortex [138]. It has been observed that GABAergic interneurons first begin a tangential route of migration at E12.5 in rodents, a time point that also happens to correspond with the early stages of neurogenesis [80,139,140]. At E12.5 in the mouse, an early population of interneurons reaches the cortex, migrating at the level of the preplate, while a second, larger proportion of interneurons migrates through the intermediate zone [139,141,142] (IZ). At a later time point of corticogenesis (E14-15), three migratory routes, called tangential migratory streams, are observed in the cortex: the marginal zone (MZ), subplate (SP), and the lower intermediate zone (IZ)/subventricular zone (SVZ) [87,142,143]. Migration is, for the most part, complete by birth, with exception of the RMS; the migration route that neurons in the RMS take to reach the olfactory bulb will not be discussed in this review. Like the processes of interneuron generation and specification, migration from the subpallial origins to the cerebral cortex is a complicated undertaking, involving the activity of various motogens, chemotactic factors, transcription factors, as well as neurotransmitters [144,145]. Each will now be briefly elaborated upon.

#### Motogens

Motogens are secreted factors that influence newly specified interneurons in their ability to migrate [141]. One such example of a motogen is Hepatocyte Growth Factor (HGF), which was discovered to regulate the migratory abilities of subpallial-derived cortical interneurons. HGF has been found to be required for interneuron migration, and encourages interneurons to migrate away from their sites of origin [146]. *mu*-*PAR* nulls (mu-PAR is a required component of the HGF pathway) demonstrate significant deficits in interneuron migration from the GEs to the cortex [146,147]. In their 2003 study Powell *et al* also presented the finding that HGF loss of function has an effect on some, but not all, subsets of interneurons [147].

In addition to HGF, the neurotrophin family of genes serve as motogens for migratory cortical interneurons. Various developmental studies have demonstrated that the cortex is positive for neurotrophin expression [148-151], and that the neurotrophin receptors TrkB and TrkC (tyrosine kinase receptors) are expressed by interneurons [152,153]. One member of the neurotrophins is known as Brain-Derived Neurotrophic Factor (BDNF) [154]. Loss of proper BDNF signaling has been shown to result in a downregulation of cortical interneuron markers [155-157].

Lastly, the neurotrophin glial cell line-derived neurotrophic factor (GDNF), which is expressed in cortical interneuron migratory routes [158], acts as a motogen for GABAergic interneurons. A 2002 study demonstrated that members of the glial derived neurotrophic factor family bind to specific types of receptors known as GFRα1-4 [159]. Loss of GFRα1 in mice manifests in improper migration from the MGE and an inability to reach the cortex compared to wild type [159]. A more recent study proposed, using *GFR*α*1* null mice, that GFRα1 signaling may serve to guide the development of a population of PV-expressing interneurons destined for the cortex [160].

#### Chemorepellants

Whereas motogens affect the ability of interneurons to migrate, chemorepellants and chemoattractants serve to provide migratory cells with the information about which *direction* to migrate. Conceivably, chemorepellants serve as a type of guidance cue that will direct migrating interneurons *away* from a certain area. Wichterle and colleagues have shown through cell culture studies that in the telencephalon, the GEs exert a repulsive force on interneurons, allowing the postulation that chemorepellants are primarily expressed in the subpallium [161].

One such example of a chemorepellant is the family of ligands known as the semaphorins (Sema) [162]. The semaphorins are able to exert their repulsive forces upon interneurons because interneurons express neuropilins (Nrp1 and Nrp2) and plexin coreceptors, both of which recognize the semaphorins that are expressed within the LGE [162]. A 2003 study demonstrated that two semaphorins in particular, Sema 3A and 3 F, regulate the migratory capacity of GABAergic interneurons toward the cortex [163]. Additionally, Zimmer *et al* have shown that the chondroitin sulfate proteoglycan known as chondroitin-4-sulfate acts in concert with Sema 3A to repel migrating cortical interneurons within the LGE, further defining the boundary of the migratory route of interneurons [164].

Slit1 is a second chemorepellant whose expression is found in the ventricular zone and the subventricular zone of the GEs, as well as the embryonic POA [165-168]. Slit1 is able to exert its chemorepulsive effects due to the expression of its receptor, Roundabout (Robo1), on migrating cortical interneurons [165-168]. Analysis of the expression patterns of both Slit and Robo proteins are indicative that interneurons are repelled from their sites of origin in the GEs [169-172].

The last type of chemorepellant that will be discussed is Ephrin and its Ephrin receptor tyrosine kinases, which are repulsive cues for MGE-derived interneurons, as demonstrated both *in vitro* and *in vivo*[173,174]. Ephrin A5 in particular is expressed in the ventricular zone of the GE, whereas its receptor, EphA4, is expressed by calbindin-expressing MGE-derived interneurons [173]. Interestingly, siRNA knockdown of the EphA4 receptor resulted in the inability of ephrin A3 to exert its repulsive effects upon interneurons within the MGE [174].

#### Chemoattractants

While inhibitory signals exhibited by chemorepellants are undoubtedly essential in defining the tangential migratory route cortical interneurons undertake, chemoatractive factors are just as important in aiding the migration process. Whereas chemorepellants are localized in the subpallium, chemoattractive molecules are present in the pallium. One such chemoattractant is the chemokine CXCL12, which carries out signaling through its receptors CXCR4 and CXCR7. Interneurons derived from the MGE are found to express both of these receptors [175]. The expression pattern of CXCL12 varies throughout development: its expression remains high in the marginal zone and subventricular zone until time point E14.5, but during later stages of corticogenesis its expression is significantly reduced in the subventricular zone (expression in the marginal zone remains unchanged) [176]. Li *et al* have described CXCL12’s function: it exerts its attractant force on MGE-derived interneurons, guiding them to the previously mentioned tangential migratory streams; these cells remain there until intracortical migrations are received [177].

The previously mentioned gene NRG1, which is a susceptibility gene in schizophrenia, also serves as a chemoattractant in the interneuronal migration process. NRG1 was discovered to be essential for interneurons to leave the MGE, traverse through the LGE and into the cortical wall [178]. Flames *et al* more specifically demonstrated that different isoforms of NRG1 possess unique migratory roles [178]. When interneurons are exposed to an exogenous source of NRG1, neurons extend new neurites in the direction of the source [179], suggesting endogenous NRG1 present during development serves the same purpose. Reduction or loss of NRG1 in the forebrain prevents GABAergic interneurons from leaving the MGE [178]. Research has also been performed on the NRG receptor ErbB4, which is expressed on migratory interneurons [180]. In mature, conditional *ErbB4* mutants, a reduced number of cortical interneurons is observed [178].

#### Transcription factors

While both motogenic and chemotactic factors have been recognized as classic influences on interneuron migration to the cortex, relatively recent studies have cited the roles of transcription factors in migration. One such transcription factor is the LIM homeodomain transcription factor Lh×6. MGE-derived interneurons that are actively migrating to the cerebral cortex express Lhx6 [143,181]. Moreover, Lh×6 is also expressed in most PV- and SST-positive cortical interneurons in mice [83]. Utilization of *Lhx6* knockdown using RNAi hindered the migration of cortical interneurons, lending strong support to the postulation that the Lh×6 gene somehow plays a role in migratory capability of interneurons [182]. Recent studies have also shown that Lh×6 may operate by promoting the expression of receptors such as ErbB4 (receptor for NRG1), CXCR4 and CXCR7 (receptors for chemokine CXCL12) [102].

While Nk×2.1 is known to be a key transcription factor for the specification of MGE-derived GABAergic interneurons [99,107], it is also suggested to play a role in migration of these interneurons toward the cortex. Nobrega-Pereira and colleagues have shown that proper migration of MGE-derived interneurons to the cortex requires downregulation of Nk×2.1 expression [183]. Further confirming this observation is the finding that ectopic expression of Nk×2.1 in migrating MGE interneuron progenitors results in the inability of Sema3A and Sema3F to exert their repulsive effects [183]. Additional studies should be performed to further elucidate the mechanism by which Nk×2.1 acts in the migration process.

The Dlx family of homeobox genes is involved in migration of interneurons to the cortex [124,184]. More specifically, Dl×1 and Dl×2 may repress the genes PAK3 and MAP2 in order to restrain neurite outgrowth [184]. PAK3 is a gene involved in regulating and maintaining cytoskeletal dynamics, and whose expression is low in migrating interneurons; *Dlx1*/*2* mutants show aberrant, increased PAK3 expression, with an inability to migrate to the cortex [184]. Reducing these aberrant levels of PAK3 restores the ability of these interneurons to migrate tangentially to the cortex [184]. Additionally, results from another study have shown that Dl×1 and Dl×2 may play a role in repression of Nrp2, the receptor for semaphorins [107], suggesting the Dlx genes have multiple modes of action to regulate migration.

Lastly,the homeobox transcription factor known as Arx is located downstream of the Dlx genes, and Dlx function has been shown to directly affect Arx expression: *Dlx1*/*2* double mutants show a down-regulation of Arx expression [185]. In the absence of Arx, MGE-derived interneuron progenitors are unable to migrate toward the cortex, resulting in reduced numbers of interneurons in the cortex [186,187]. Additionally, mice possessing conditional *Arx* mutations also have reduced cortical interneuron numbers and have epilepsy [188], demonstrating the importance of this transcription factor in interneuron specification within the MGE.

#### Neurotransmitters

While their role in migration toward the cerebral cortex may seem unconventional, evidence is emerging that neurotransmitters do indeed aid GABAergic interneurons in their movement toward the cortex. The two major neurotransmitters that will be elaborated upon are GABA and dopamine. Migrating interneurons express the GABA_A_ and GABA_B_, two main receptors for GABA [189-191]. Reducing GABA activity via neutralizing antibodies results in an accumulation of interneurons at the corticostriatal junction, and prevents their entry into the cortical wall [189]. A more recent study also showed that migrating interneurons have a higher affinity for GABA, relative to non-migrating interneurons that are localized in the MGE [191].

Like the GABA receptors, migrating cortical interneurons also express D_1_ and D_2_, which are the receptors for dopamine [192,193]. D_1_ and D_2_ receptors, when activated, are known to have opposite functions from one another [192]. Generation of individual knockouts further confirms the inverse function of these receptors: *D*_*1*_ nulls possess a decreased ability to migrate, allowing the conclusion that D_1_ normally functions to promote cortical interneuron migration. On the other hand, *D*_*2*_ knockouts possess increased migratory capability, suggesting that its role is to inhibit migration of interneurons [192]. It is clear that additional studies must be performed to further elucidate the roles that neurotransmitters such as GABA and dopamine play in the migratory process, as the mechanisms by which they operate are still unknown.

Interestingly enough, while there are clearly many factors that influence the migratory route of GABAergic interneurons to the cortex, Sahara *et al* have demonstrated that the proportion of cortical interneurons, relative to excitatory neurons, stays the same from the beginning of neurogenesis until adulthood [194]. Data from this study suggests that approximately 1 in 5 interneurons migrating tangentially toward the cerebral cortex in mice are GAD67-positive, indicating they are inhibitory GABAergic interneurons [194].

It is important to note that once GABAergic interneurons successfully reach the cortex, a process referred to as “intracortical migration” must be carried out, whereby interneurons undergo different migratory routes within the cortex to reach their ultimate destinations. This process, as well as interneuron lamination, is beyond the scope of this review and will not be discussed. For a detailed discussion of these processes, refer to the review by Faux *et al*[195].

To date, the molecular mechanisms that regulate the termination of interneuron migration in the cortex are largely unknown. However, Bortone and Polleux have shown that the potassium-chloride cotransporter KCC2 plays an integral role in terminating interneuron migration [196]. When interneurons are actively migrating, ambient GABA and glutamate initially stimulate the motility through activation of GABA_A_ and AMPA/NMDA receptors. However, once interneurons reach the cortex, upregulation of KCC2 results in a hyperpolarization of membrane potential, thereby serving as a stop signal that interneurons are able to sense [196]. Further investigations should be performed to further elucidate the mechanisms underlying termination of migration.

### GABAergic dysfunction and neuropsychiatric disorders

As can be imagined, there is a delicate balance between excitatory and inhibitory inputs that must be carefully maintained with regard to cortical circuits. It is thus conceivable that a number of neuropsychiatric disorders may stem from an alteration or disruption in the balance of excitation vs. inhibition in the cortical circuitry. Additionally, a number of studies have shown that the lack of proper cortical interneuron specification may play a significant role in the development of neurological disorders. This may entail a deviation from either the course of interneuron development, or aberrant transcriptional regulation in the cortical interneuron specification process. Schizophrenia, a severe mental illness, will be utilized as an example of cortical circuitry gone awry. Understanding the effects of both GABAergic neurotransmission, alterations in inhibitory cortical circuits, and how they may be responsible for the clinical features observed in schizophrenia are paramount to this field of research.

Schizophrenia is a mental disorder that is characterized by a variety of symptoms, with cognitive deficits being recognized as both the core and enduring features of this illness [197]. Hallmark features of schizophrenia include auditory hallucinations, paranoid or bizarre delusions, disorganized speech and thinking, and social withdrawal. Additionally, working memory and attention are characteristically impaired in schizophrenic patients [197]. Onset of symptoms occurs in young adulthood. While environmental influences are widely suggested to play a contributory role in development of this illness, genetics and familial predisposition play a very significant role: a meta-analytic review on cognitive performance between relatives of schizophrenic patients and healthy control subjects demonstrated that the same cognitive deficits found in patients with schizophrenia are also found in non-affected relatives [198].

It was initially postulated that schizophrenic patients demonstrating GABAergic interneuron deficits had a significantly reduced number of interneurons relative to non-schizophrenic subjects [199]. While this may still hold true, this field of research is seeing a gradual shift toward the belief that GABAergic dysfunction in schizophrenia may be a result of disruption or imbalance of inhibitory neurocircuitry, rather than a sheer reduction in neuron number [9].

A common trend observed in independently performed studies has shown that only certain interneuronal subtypes seem to be affected in schizophrenia. Schizophrenic patients showed a marked reduction of GAD67 mRNA levels in PV-expressing interneurons of the prefrontal cortex, while GAD67 mRNA levels did not differ in CR-positive interneurons of schizophrenics and healthy controls [200,201]. A number of studies have postulated that the population of PV-expressing GABAergic interneurons in people with schizophrenia may not be functioning at full capacity, and this may contribute to the cognitive deficits observed with this illness. An inability of PV-positive interneurons to function properly may result in disruption of inhibitory input onto pyramidal neurons, and impairment of synchronization in the gamma range [202,203]. This theory is corroborated by the observation that schizophrenic patients, when asked to do working memory tasks, display abnormal gamma frequency oscillations in the prefrontal cortex relative to healthy, non-schizophrenic subjects [204-207].

With the knowledge that the parvalbumin-expressing subtype of GABAergic interneurons in particular is largely affected in schizophrenia, it is sensible to seek out factors that play a role in the development of the PV-positive subtype. One such protein is the receptor tyrosine protein kinase ErbB4, which is a receptor for the trophic factor Neuregulin 1 (NRG1). Research was initially focused on NRG1, which was first identified as a susceptibility gene for schizophrenia in an Icelandic population, and then confirmed as a susceptibility gene in an unrelated Scottish population [208]. NRG1 plays a variety of roles during neural development, including modulation of neuronal migration, synaptogenesis, gliogenesis, myelination, and neurotransmission [209]. In particular, NRG1 stimulates GABA release from interneurons, which inhibits pyramidal cells in the prefrontal cortex [210]. It can be imagined that disruption of NRG1 function can have greatly affect the balance between excitatory and inhibitory input. The possible mechanisms by which altered function of NRG1 and its receptor ErbB4 contribute to schizophrenia have been reviewed by Mei and Xiong [211].

With regard to schizophrenia, attention is shifted to NRG1’s receptor, ErbB4: this is largely due to the fact that ErbB4 is a receptor preferentially expressed by interneurons migrating tangentially from the ventral to the dorsal telencephalon [180] and by both embryonic and postnatal PV-expressing interneurons with chandelier and basket cell morphology [212,213]. Importantly, *in vitro* and *in vivo* gain- and loss-of-function experiments prove that ErbB4 promotes the formation of axo-axonic inhibitory synapses over pyramidal neurons in a cell-autonomous manner [212]. More evidence suggests that ErbB4 exerts its effects on PV-positive interneurons: PV-*ErbB4*^-/-^ mice exhibit a schizophrenia-like phenotype much like that of *NRG1*-*null* and *ErbB4*-*null* mutants; these mice are hyperactive and show impaired working memory [210]. Deletion of ErbB4 in PV-positive interneurons in these mice also results in fewer synapses being made onto pyramidal neurons [210], thus demonstrating the importance of this transmembrane receptor (as well as Neuregulin 1) in affecting the proper development of at least one subset of GABAergic interneurons.

An additional gene whose disruption predisposes to schizophrenia was first identified in 2000 in a large Scottish family, and named Disrupted-In-Schizophrenia 1 (DISC1) [214]. Utilization of DISC1 genetically engineered mice served as a model for mental illnesses such as schizophrenia, and analysis of these mice showed that dominant-negative *DISC1* mice display behavioral abnormalities and a depression-like deficit [215]. Hikida *et al* also importantly show that DSC1 plays a role in the development of PV-expressing cortical interneurons: dominant-negative *DISC1* mice possess pyramidal cells with reduced PV immunoreactivity in the cortex [215]. Another study using knockdown of *DISC1* has generated the same results regarding decreased PV immunoreactivity [216]. *DISC1* knockdown in prefrontal cortex pyramidal neurons during the pre- and perinatal stages results in abnormal postnatal mesocortical dopaminergic maturation, as well as behavioral abnormalities linked to disrupted cortical circuitry during adulthood [216]. A recent review by Porteus *et al* offers a detailed summary of the progress that several research groups have made in understanding the role DISC1 protein plays in neurosignaling and neurodevelopment since its initial discovery [217]. While the specific role that DISC1 plays in maintaining cortical interneuron development has yet to be elucidated, it is clear that it is needed to allow proper development of PV-expressing interneurons.

Dysbindin is another schizophrenia susceptibility gene, which plays a role in dopamine receptor trafficking [218]. Multipoint linkage analysis in an Irish population showing genetic variation in the 6p22.3 gene DTNBP1 (dystrobrevin-binding protein 1, the human ortholog of dysbindin) was found to be associated with schizophrenia, thereby spurring a number of investigations of this gene [219]. Findings from a subsequent study indicated that reduction in DTNBP1, frequently observed in schizophrenia, was linked to glutamatergic alterations in intrinsic hippocampal formation connections [220]. Another study corroborated the finding of dysbindin reduction: schizophrenic patients show decreased levels of dysbindin mRNA in multiple layers of the dorsolateral prefrontal cortex relative to healthy subjects [221]. Importantly, *Dtnbp1*-knockout mice possess cortical and striatal PV-expressing, fast-spiking interneurons with a significant reduction in excitability [218]. This therefore results in decreased inhibitory input to pyramidal neurons in layer V of the prefrontal cortex [218].

While it was initially believed that DISC1 and dysbindin both served as independent susceptibility genes to schizophrenia, a 2011 study investigated whether both DISC1 and dysbindin proteins converged onto a common pathway. Co-aggregation of the two proteins in postmortem brains of patients with mental disease, but not that of healthy patients, was observed, demonstrating that DISC1 and dysbindin do indeed directly interact with each other on a molecular level [222]. It is clear that there are many genetic components implicated in the development of schizophrenia, each of which serve to somehow tip the excitatory-inhibitory balance within the neural circuitry. It is also clear how important of a role GABAergic interneurons play in maintaining this balance. It is important, however, to remember that this is merely the tip of the iceberg – there are many other neuropsychiatric disorders such as epilepsy, autism spectrum disorders, and various intellectual disabilities, many of which are reviewed extensively in a review by the Marin research group [9], that possess some sort of alteration or disruption of balance within the developing nervous system.

### Summary

The population of GABAergic interneurons in the cerebral cortex is a clearly a diverse one, comprised of many different subtypes and functions. While each interneuronal subtype is characteristically unique with regard to function, electrophysiology, immunohistochemical profile, axonal targeting and firing pattern, the overlapping features between particular subtypes makes the method of categorizing each subset of GABAergic interneurons quite challenging. Most recent undertakings in the study of cortical interneurons have been concerned with constructing the transcriptional network of genes that are involved in the specification of these interneurons, in pre-migratory stages within the subpallium as well as during the migratory phase of interneuron precursors. Additional investigations must be performed in order to understand the specific transcription factors and signaling pathways involved in the specification of interneuronal fate. More specifically, studies must be undertaken to understand how the 5HT3aR-expressing interneuron lineage is specified before migration of these interneuronal progenitors to the cerebral cortex, as it is such a large proportion of the interneuron population. Additionally, not enough is known about the differential specification of PV- and SST-positive subgroups within the Nkx2.1-expressing lineage of interneuronal progenitors. The emergence of cutting edge genetic technologies should be utilized to target specific interneuronal subtypes and understand what key players will determine their fate and function. On a broader level, investigations must also be carried out to understand the differences in origin and specification of rodent vs. human GABAergic interneurons. Understanding the mechanisms behind human GABAergic interneuron specification may enable a large step forward in treating various neurological disorders linked to alterations/dysfunctions in interneuron populations. An increased understanding of the mechanism(s) by which GABAergic interneurons are able to integrate properly and seamlessly into the cortex after migration is complete is also warranted.

## Competing interests

The authors declare that they have no competing interests.

## Authors’ contribution

Both CK and WL contributed to writing of the manuscript. All authors read and approved the final manuscript.

## References

[B1] Hensch TK Critical period plasticity in local cortical circuits Nat Rev Neurosci 2005 6 11 877 888 10.1038/nrn1787 16261181

[B2] Owens DF Kriegstein AR Is there more to GABA than synaptic inhibition? Nat Rev Neurosci 2002 3 9 715 727 10.1038/nrn919 12209120

[B3] Wang XJ Tegner J Constantinidis C Goldman-Rakic PS Division of labor among distinct subtypes of inhibitory neurons in a cortical microcircuit of working memory Proc Natl Acad Sci U S A 2004 101 5 1368 1373 10.1073/pnas.0305337101 14742867 PMC337059

[B4] Whittington MA Traub RD Interneuron diversity series: inhibitory interneurons and network oscillations in vitro Trends Neurosci 2003 26 12 676 682 10.1016/j.tins.2003.09.016 14624852

[B5] Dreifuss JJ Kelly JS Krnjevic K Cortical inhibition and gamma-aminobutyric acid Exp Brain Res 1969 9 2 137 154 10.1007/BF00238327 5346460

[B6] Fonnum F Storm-Mathisen J GABA synthesis in rat hippocampus correlated to the distribution of inhibitory neurons Acta Physiol Scand 1969 76 1 35A 36A 5823399

[B7] Somogyi P Freund TF Wu JY Smith AD The section-Golgi impregnation procedure. 2. Immunocytochemical demonstration of glutamate decarboxylase in Golgi-impregnated neurons and in their afferent synaptic boutons in the visual cortex of the cat Neuroscience 1983 9 3 475 490 10.1016/0306-4522(83)90167-7 6194475

[B8] Rudy B Fishell G Lee S Hjerling-Leffler J Three groups of interneurons account for nearly 100% of neocortical GABAergic neurons Dev Neurobiol 2011 71 1 45 61 10.1002/dneu.20853 21154909 PMC3556905

[B9] Marin O Interneuron dysfunction in psychiatric disorders Nat Rev Neurosci 2012 13 2 107 120 22251963 10.1038/nrn3155

[B10] Gibson JR Beierlein M Connors BW Two networks of electrically coupled inhibitory neurons in neocortex Nature 1999 402 6757 75 79 10.1038/47035 10573419

[B11] Porter JT Johnson CK Agmon A Diverse types of interneurons generate thalamus-evoked feedforward inhibition in the mouse barrel cortex J Neurosci 2001 21 8 2699 2710 11306623 10.1523/JNEUROSCI.21-08-02699.2001 PMC6762510

[B12] Berger TK Silberberg G Perin R Markram H Brief bursts self-inhibit and correlate the pyramidal network PLoS Biol 2010 8 9 e1000473 10.1371/journal.pbio.1000473 20838653 PMC2935452

[B13] Silberberg G Gupta A Markram H Stereotypy in neocortical microcircuits Trends Neurosci 2002 25 5 227 230 10.1016/S0166-2236(02)02151-3 11972952

[B14] Wang Y Toledo-Rodriguez M Gupta A Wu C Silberberg G Luo J Markram H Anatomical, physiological and molecular properties of Martinotti cells in the somatosensory cortex of the juvenile rat J Physiol 2004 561 Pt 1 65 90 15331670 10.1113/jphysiol.2004.073353 PMC1665344

[B15] Somogyi P Tamas G Lujan R Buhl EH Salient features of synaptic organisation in the cerebral cortex Brain Res Brain Res Rev 1998 26 2–3 113 135 9651498 10.1016/s0165-0173(97)00061-1

[B16] Ascoli GA Alonso-Nanclares L Anderson SA Barrionuevo G Benavides-Piccione R Burkhalter A Buzsaki G Cauli B Defelipe J Fairen A Petilla terminology: nomenclature of features of GABAergic interneurons of the cerebral cortex Nat Rev Neurosci 2008 9 7 557 568 18568015 10.1038/nrn2402 PMC2868386

[B17] Cauli B Audinat E Lambolez B Angulo MC Ropert N Tsuzuki K Hestrin S Rossier J Molecular and physiological diversity of cortical nonpyramidal cells J Neurosci 1997 17 10 3894 3906 9133407 10.1523/JNEUROSCI.17-10-03894.1997 PMC6573690

[B18] DeFelipe J Neocortical neuronal diversity: chemical heterogeneity revealed by colocalization studies of classic neurotransmitters, neuropeptides, calcium-binding proteins, and cell surface molecules Cereb Cortex 1993 3 4 273 289 10.1093/cercor/3.4.273 8104567

[B19] DeFelipe J Hendry SH Jones EG Visualization of chandelier cell axons by parvalbumin immunoreactivity in monkey cerebral cortex Proc Natl Acad Sci U S A 1989 86 6 2093 2097 10.1073/pnas.86.6.2093 2648389 PMC286854

[B20] Gonchar Y Burkhalter A Three distinct families of GABAergic neurons in rat visual cortex Cereb Cortex 1997 7 4 347 358 10.1093/cercor/7.4.347 9177765

[B21] Hendry SH Jones EG Emson PC Lawson DE Heizmann CW Streit P Two classes of cortical GABA neurons defined by differential calcium binding protein immunoreactivities Exp Brain Res 1989 76 2 467 472 2767197 10.1007/BF00247904

[B22] Kawaguchi Y Kubota Y GABAergic cell subtypes and their synaptic connections in rat frontal cortex Cereb Cortex 1997 7 6 476 486 10.1093/cercor/7.6.476 9276173

[B23] Markram H Toledo-Rodriguez M Wang Y Gupta A Silberberg G Wu C Interneurons of the neocortical inhibitory system Nat Rev Neurosci 2004 5 10 793 807 10.1038/nrn1519 15378039

[B24] Somogyi P Klausberger T Defined types of cortical interneurone structure space and spike timing in the hippocampus J Physiol 2005 562 Pt 1 9 26 15539390 10.1113/jphysiol.2004.078915 PMC1665488

[B25] Lee S Hjerling-Leffler J Zagha E Fishell G Rudy B The largest group of superficial neocortical GABAergic interneurons expresses ionotropic serotonin receptors J Neurosci 2010 30 50 16796 16808 10.1523/JNEUROSCI.1869-10.2010 21159951 PMC3025500

[B26] Xu X Callaway EM Laminar specificity of functional input to distinct types of inhibitory cortical neurons J Neurosci 2009 29 1 70 85 10.1523/JNEUROSCI.4104-08.2009 19129386 PMC2656387

[B27] Connors BW Gutnick MJ Intrinsic firing patterns of diverse neocortical neurons Trends Neurosci 1990 13 3 99 104 10.1016/0166-2236(90)90185-D 1691879

[B28] Goldberg EM Clark BD Zagha E Nahmani M Erisir A Rudy B K + channels at the axon initial segment dampen near-threshold excitability of neocortical fast-spiking GABAergic interneurons Neuron 2008 58 3 387 400 10.1016/j.neuron.2008.03.003 18466749 PMC2730466

[B29] Pinto DJ Brumberg JC Simons DJ Circuit dynamics and coding strategies in rodent somatosensory cortex J Neurophysiol 2000 83 3 1158 1166 10712446 10.1152/jn.2000.83.3.1158

[B30] Miller LM Escabi MA Schreiner CE Feature selectivity and interneuronal cooperation in the thalamocortical system J Neurosci 2001 21 20 8136 8144 11588186 10.1523/JNEUROSCI.21-20-08136.2001 PMC6763836

[B31] Pouille F Scanziani M Enforcement of temporal fidelity in pyramidal cells by somatic feed-forward inhibition Science 2001 293 5532 1159 1163 10.1126/science.1060342 11498596

[B32] Pinto DJ Hartings JA Brumberg JC Simons DJ Cortical damping: analysis of thalamocortical response transformations in rodent barrel cortex Cereb Cortex 2003 13 1 33 44 10.1093/cercor/13.1.33 12466213

[B33] Lawrence JJ McBain CJ Interneuron diversity series: containing the detonation–feedforward inhibition in the CA3 hippocampus Trends Neurosci 2003 26 11 631 640 10.1016/j.tins.2003.09.007 14585604

[B34] Gabernet L Jadhav SP Feldman DE Carandini M Scanziani M Somatosensory integration controlled by dynamic thalamocortical feed-forward inhibition Neuron 2005 48 2 315 327 10.1016/j.neuron.2005.09.022 16242411

[B35] Cruikshank SJ Lewis TJ Connors BW Synaptic basis for intense thalamocortical activation of feedforward inhibitory cells in neocortex Nat Neurosci 2007 10 4 462 468 17334362 10.1038/nn1861

[B36] Hasenstaub A Shu Y Haider B Kraushaar U Duque A McCormick DA Inhibitory postsynaptic potentials carry synchronized frequency information in active cortical networks Neuron 2005 47 3 423 435 10.1016/j.neuron.2005.06.016 16055065

[B37] Haider B McCormick DA Rapid neocortical dynamics: cellular and network mechanisms Neuron 2009 62 2 171 189 10.1016/j.neuron.2009.04.008 19409263 PMC3132648

[B38] Woodruff A Xu Q Anderson SA Yuste R Depolarizing effect of neocortical chandelier neurons Frontiers in neural circuits 2009 3 15 19876404 10.3389/neuro.04.015.2009 PMC2769545

[B39] Szabadics J Varga C Molnar G Olah S Barzo P Tamas G Excitatory effect of GABAergic axo-axonic cells in cortical microcircuits Science 2006 311 5758 233 235 10.1126/science.1121325 16410524

[B40] Glickfeld LL Roberts JD Somogyi P Scanziani M Interneurons hyperpolarize pyramidal cells along their entire somatodendritic axis Nat Neurosci 2009 12 1 21 23 10.1038/nn.2230 19029887 PMC3505023

[B41] Blatow M Rozov A Katona I Hormuzdi SG Meyer AH Whittington MA Caputi A Monyer H A novel network of multipolar bursting interneurons generates theta frequency oscillations in neocortex Neuron 2003 38 5 805 817 10.1016/S0896-6273(03)00300-3 12797964

[B42] Uematsu M Hirai Y Karube F Ebihara S Kato M Abe K Obata K Yoshida S Hirabayashi M Yanagawa Y Quantitative chemical composition of cortical GABAergic neurons revealed in transgenic venus-expressing rats Cereb Cortex 2008 18 2 315 330 17517679 10.1093/cercor/bhm056

[B43] Beierlein M Gibson JR Connors BW Two dynamically distinct inhibitory networks in layer 4 of the neocortex J Neurophysiol 2003 90 5 2987 3000 10.1152/jn.00283.2003 12815025

[B44] Kapfer C Glickfeld LL Atallah BV Scanziani M Supralinear increase of recurrent inhibition during sparse activity in the somatosensory cortex Nat Neurosci 2007 10 6 743 753 10.1038/nn1909 17515899 PMC3518866

[B45] Reyes A Lujan R Rozov A Burnashev N Somogyi P Sakmann B Target-cell-specific facilitation and depression in neocortical circuits Nat Neurosci 1998 1 4 279 285 10.1038/1092 10195160

[B46] Silberberg G Markram H Disynaptic inhibition between neocortical pyramidal cells mediated by Martinotti cells Neuron 2007 53 5 735 746 10.1016/j.neuron.2007.02.012 17329212

[B47] Fanselow EE Richardson KA Connors BW Selective, state-dependent activation of somatostatin-expressing inhibitory interneurons in mouse neocortex J Neurophysiol 2008 100 5 2640 2652 10.1152/jn.90691.2008 18799598 PMC2585405

[B48] Ma Y Hu H Berrebi AS Mathers PH Agmon A Distinct subtypes of somatostatin-containing neocortical interneurons revealed in transgenic mice J Neurosci 2006 26 19 5069 5082 10.1523/JNEUROSCI.0661-06.2006 16687498 PMC2020857

[B49] McGarry LM Packer AM Fino E Nikolenko V Sippy T Yuste R Quantitative classification of somatostatin-positive neocortical interneurons identifies three interneuron subtypes Front Neural Circ 2010 4 12 10.3389/fncir.2010.00012 PMC2896209 20617186

[B50] Xu X Roby KD Callaway EM Mouse cortical inhibitory neuron type that coexpresses somatostatin and calretinin J Comp Neurol 2006 499 1 144 160 10.1002/cne.21101 16958092

[B51] Zimmer G Rudolph J Landmann J Gerstmann K Steinecke A Gampe C Bolz J Bidirectional ephrinB3/EphA4 signaling mediates the segregation of medial ganglionic eminence- and preoptic area-derived interneurons in the deep and superficial migratory stream J Neurosci 2011 31 50 18364 18380 10.1523/JNEUROSCI.4690-11.2011 22171039 PMC6623906

[B52] Gonchar Y Wang Q Burkhalter A Multiple distinct subtypes of GABAergic neurons in mouse visual cortex identified by triple immunostaining Front Neuroanat 2007 1 3 18958197 10.3389/neuro.05.003.2007 PMC2525923

[B53] Miyoshi G Butt SJ Takebayashi H Fishell G Physiologically distinct temporal cohorts of cortical interneurons arise from telencephalic Olig2-expressing precursors J Neurosci 2007 27 29 7786 7798 10.1523/JNEUROSCI.1807-07.2007 17634372 PMC6672881

[B54] Xu X Roby KD Callaway EM Immunochemical characterization of inhibitory mouse cortical neurons: three chemically distinct classes of inhibitory cells J Comp Neurol 2010 518 3 389 404 10.1002/cne.22229 19950390 PMC2804902

[B55] Cauli B Porter JT Tsuzuki K Lambolez B Rossier J Quenet B Audinat E Classification of fusiform neocortical interneurons based on unsupervised clustering Proc Natl Acad Sci U S A 2000 97 11 6144 6149 10.1073/pnas.97.11.6144 10823957 PMC18572

[B56] Miyoshi G Hjerling-Leffler J Karayannis T Sousa VH Butt SJ Battiste J Johnson JE Machold RP Fishell G Genetic fate mapping reveals that the caudal ganglionic eminence produces a large and diverse population of superficial cortical interneurons J Neurosci 2010 30 5 1582 1594 10.1523/JNEUROSCI.4515-09.2010 20130169 PMC2826846

[B57] David C Schleicher A Zuschratter W Staiger JF The innervation of parvalbumin-containing interneurons by VIP-immunopositive interneurons in the primary somatosensory cortex of the adult rat Eur J Neurosci 2007 25 8 2329 2340 10.1111/j.1460-9568.2007.05496.x 17445231

[B58] Acsady L Gorcs TJ Freund TF Different populations of vasoactive intestinal polypeptide-immunoreactive interneurons are specialized to control pyramidal cells or interneurons in the hippocampus Neuroscience 1996 73 2 317 334 10.1016/0306-4522(95)00609-5 8783252

[B59] Ferezou I Cauli B Hill EL Rossier J Hamel E Lambolez B 5-HT3 receptors mediate serotonergic fast synaptic excitation of neocortical vasoactive intestinal peptide/cholecystokinin interneurons J Neurosci 2002 22 17 7389 7397 12196560 10.1523/JNEUROSCI.22-17-07389.2002 PMC6757992

[B60] Galarreta M Erdelyi F Szabo G Hestrin S Electrical coupling among irregular-spiking GABAergic interneurons expressing cannabinoid receptors J Neurosci 2004 24 44 9770 9778 10.1523/JNEUROSCI.3027-04.2004 15525762 PMC6730255

[B61] Porter JT Cauli B Staiger JF Lambolez B Rossier J Audinat E Properties of bipolar VIPergic interneurons and their excitation by pyramidal neurons in the rat neocortex Eur J Neurosci 1998 10 12 3617 3628 10.1046/j.1460-9568.1998.00367.x 9875341

[B62] Caputi A Rozov A Blatow M Monyer H Two calretinin-positive GABAergic cell types in layer 2/3 of the mouse neocortex provide different forms of inhibition Cereb Cortex 2009 19 6 1345 1359 10.1093/cercor/bhn175 18842664

[B63] Butt SJ Fuccillo M Nery S Noctor S Kriegstein A Corbin JG Fishell G The temporal and spatial origins of cortical interneurons predict their physiological subtype Neuron 2005 48 4 591 604 10.1016/j.neuron.2005.09.034 16301176

[B64] Kawaguchi Y Kubota Y Physiological and morphological identification of somatostatin- or vasoactive intestinal polypeptide-containing cells among GABAergic cell subtypes in rat frontal cortex J Neurosci 1996 16 8 2701 2715 8786446 10.1523/JNEUROSCI.16-08-02701.1996 PMC6578756

[B65] Olah S Komlosi G Szabadics J Varga C Toth E Barzo P Tamas G Output of neurogliaform cells to various neuron types in the human and rat cerebral cortex Front Neural Circ 2007 1 4 10.3389/neuro.04.004.2007 PMC2526278 18946546

[B66] Price CJ Cauli B Kovacs ER Kulik A Lambolez B Shigemoto R Capogna M Neurogliaform neurons form a novel inhibitory network in the hippocampal CA1 area J Neurosci 2005 25 29 6775 6786 10.1523/JNEUROSCI.1135-05.2005 16033887 PMC6725364

[B67] Simon A Olah S Molnar G Szabadics J Tamas G Gap-junctional coupling between neurogliaform cells and various interneuron types in the neocortex J Neurosci 2005 25 27 6278 6285 10.1523/JNEUROSCI.1431-05.2005 16000617 PMC6725286

[B68] Zsiros V Maccaferri G Electrical coupling between interneurons with different excitable properties in the stratum lacunosum-moleculare of the juvenile CA1 rat hippocampus J Neurosci 2005 25 38 8686 8695 10.1523/JNEUROSCI.2810-05.2005 16177037 PMC6725508

[B69] Tamas G Lorincz A Simon A Szabadics J Identified sources and targets of slow inhibition in the neocortex Science 2003 299 5614 1902 1905 10.1126/science.1082053 12649485

[B70] Corbin JG Butt SJ Developmental mechanisms for the generation of telencephalic interneurons Dev Neurobiol 2011 71 8 710 732 10.1002/dneu.20890 21485015

[B71] O’Rahilly R Gardner E The initial development of the human brain Acta Anat 1979 104 2 123 133 10.1159/000145061 442966

[B72] Van Eden CG Mrzljak L Voorn P Uylings HB Prenatal development of GABA-ergic neurons in the neocortex of the rat J Comp Neurol 1989 289 2 213 227 10.1002/cne.902890204 2808764

[B73] DeDiego I Smith-Fernandez A Fairen A Cortical cells that migrate beyond area boundaries: characterization of an early neuronal population in the lower intermediate zone of prenatal rats Eur J Neurosci 1994 6 6 983 997 10.1111/j.1460-9568.1994.tb00593.x 7952285

[B74] de Carlos JA Lopez-Mascaraque L Valverde F Dynamics of cell migration from the lateral ganglionic eminence in the rat J Neurosci 1996 16 19 6146 6156 8815897 10.1523/JNEUROSCI.16-19-06146.1996 PMC6579193

[B75] Tamamaki N Fujimori KE Takauji R Origin and route of tangentially migrating neurons in the developing neocortical intermediate zone J Neurosci 1997 17 21 8313 8323 9334406 10.1523/JNEUROSCI.17-21-08313.1997 PMC6573720

[B76] Mione MC Danevic C Boardman P Harris B Parnavelas JG Lineage analysis reveals neurotransmitter (GABA or glutamate) but not calcium-binding protein homogeneity in clonally related cortical neurons J Neurosci 1994 14 1 107 123 7904303 10.1523/JNEUROSCI.14-01-00107.1994 PMC6576863

[B77] Parnavelas JG Barfield JA Franke E Luskin MB Separate progenitor cells give rise to pyramidal and nonpyramidal neurons in the rat telencephalon Cereb Cortex 1991 1 6 463 468 10.1093/cercor/1.6.463 1822752

[B78] Rakic P Lombroso PJ Development of the cerebral cortex: I. Forming the cortical structure J Am Acad Child Adolesc Psychiatry 1998 37 1 116 117 10.1097/00004583-199801000-00026 9444908

[B79] Pleasure SJ Anderson S Hevner R Bagri A Marin O Lowenstein DH Rubenstein JL Cell migration from the ganglionic eminences is required for the development of hippocampal GABAergic interneurons Neuron 2000 28 3 727 740 10.1016/S0896-6273(00)00149-5 11163262

[B80] Wonders CP Anderson SA The origin and specification of cortical interneurons Nat Rev Neurosci 2006 7 9 687 696 10.1038/nrn1954 16883309

[B81] Wonders CP Taylor L Welagen J Mbata IC Xiang JZ Anderson SA A spatial bias for the origins of interneuron subgroups within the medial ganglionic eminence Dev Biol 2008 314 1 127 136 10.1016/j.ydbio.2007.11.018 18155689 PMC2727678

[B82] Xu Q Cobos I De La Cruz E Rubenstein JL Anderson SA Origins of cortical interneuron subtypes J Neurosci 2004 24 11 2612 2622 10.1523/JNEUROSCI.5667-03.2004 15028753 PMC6729522

[B83] Cobos I Calcagnotto ME Vilaythong AJ Thwin MT Noebels JL Baraban SC Rubenstein JL Mice lacking Dlx1 show subtype-specific loss of interneurons, reduced inhibition and epilepsy Nat Neurosci 2005 8 8 1059 1068 10.1038/nn1499 16007083

[B84] Flames N Pla R Gelman DM Rubenstein JL Puelles L Marin O Delineation of multiple subpallial progenitor domains by the combinatorial expression of transcriptional codes J Neurosci 2007 27 36 9682 9695 10.1523/JNEUROSCI.2750-07.2007 17804629 PMC4916652

[B85] Wichterle H Turnbull DH Nery S Fishell G Alvarez-Buylla A In utero fate mapping reveals distinct migratory pathways and fates of neurons born in the mammalian basal forebrain Development 2001 128 19 3759 3771 11585802 10.1242/dev.128.19.3759

[B86] Corbin JG Rutlin M Gaiano N Fishell G Combinatorial function of the homeodomain proteins Nkx2.1 and Gsh2 in ventral telencephalic patterning Development 2003 130 20 4895 4906 10.1242/dev.00717 12930780

[B87] Anderson SA Marin O Horn C Jennings K Rubenstein JL Distinct cortical migrations from the medial and lateral ganglionic eminences Development 2001 128 3 353 363 11152634 10.1242/dev.128.3.353

[B88] Nery S Corbin JG Fishell G Dlx2 progenitor migration in wild type and Nkx2.1 mutant telencephalon Cereb Cortex 2003 13 9 895 903 10.1093/cercor/13.9.895 12902388

[B89] Nery S Fishell G Corbin JG The caudal ganglionic eminence is a source of distinct cortical and subcortical cell populations Nat Neurosci 2002 5 12 1279 1287 10.1038/nn971 12411960

[B90] Chameau P Inta D Vitalis T Monyer H Wadman WJ van Hooft JA The N-terminal region of reelin regulates postnatal dendritic maturation of cortical pyramidal neurons Proc Natl Acad Sci U S A 2009 106 17 7227 7232 10.1073/pnas.0810764106 19366679 PMC2678467

[B91] Inta D Alfonso J von Engelhardt J Kreuzberg MM Meyer AH van Hooft JA Monyer H Neurogenesis and widespread forebrain migration of distinct GABAergic neurons from the postnatal subventricular zone Proc Natl Acad Sci U S A 2008 105 52 20994 20999 10.1073/pnas.0807059105 19095802 PMC2605417

[B92] Vucurovic K Gallopin T Ferezou I Rancillac A Chameau P van Hooft JA Geoffroy H Monyer H Rossier J Vitalis T Serotonin 3A receptor subtype as an early and protracted marker of cortical interneuron subpopulations Cereb Cortex 2010 20 10 2333 2347 10.1093/cercor/bhp310 20083553 PMC2936799

[B93] Gelman D Griveau A Dehorter N Teissier A Varela C Pla R Pierani A Marin O A wide diversity of cortical GABAergic interneurons derives from the embryonic preoptic area J Neurosci 2011 31 46 16570 16580 10.1523/JNEUROSCI.4068-11.2011 22090484 PMC6633309

[B94] Wichterle H Garcia-Verdugo JM Herrera DG Alvarez-Buylla A Young neurons from medial ganglionic eminence disperse in adult and embryonic brain Nat Neurosci 1999 2 5 461 466 10.1038/8131 10321251

[B95] Sussel L Marin O Kimura S Rubenstein JL Loss of Nkx2.1 homeobox gene function results in a ventral to dorsal molecular respecification within the basal telencephalon: evidence for a transformation of the pallidum into the striatum Development 1999 126 15 3359 3370 10393115 10.1242/dev.126.15.3359

[B96] Jimenez D Lopez-Mascaraque LM Valverde F De Carlos JA Tangential migration in neocortical development Dev Biol 2002 244 1 155 169 10.1006/dbio.2002.0586 11900465

[B97] Azim E Jabaudon D Fame RM Macklis JD SOX6 controls dorsal progenitor identity and interneuron diversity during neocortical development Nat Neurosci 2009 12 10 1238 1247 10.1038/nn.2387 19657336 PMC2903203

[B98] Batista-Brito R Rossignol E Hjerling-Leffler J Denaxa M Wegner M Lefebvre V Pachnis V Fishell G The cell-intrinsic requirement of Sox6 for cortical interneuron development Neuron 2009 63 4 466 481 10.1016/j.neuron.2009.08.005 19709629 PMC2773208

[B99] Butt SJ Sousa VH Fuccillo MV Hjerling-Leffler J Miyoshi G Kimura S Fishell G The requirement of Nkx2-1 in the temporal specification of cortical interneuron subtypes Neuron 2008 59 5 722 732 10.1016/j.neuron.2008.07.031 18786356 PMC2562525

[B100] Liodis P Denaxa M Grigoriou M Akufo-Addo C Yanagawa Y Pachnis V Lhx6 activity is required for the normal migration and specification of cortical interneuron subtypes J Neurosci 2007 27 12 3078 3089 10.1523/JNEUROSCI.3055-06.2007 17376969 PMC6672459

[B101] Wang Y Dye CA Sohal V Long JE Estrada RC Roztocil T Lufkin T Deisseroth K Baraban SC Rubenstein JL Dlx5 and Dlx6 regulate the development of parvalbumin-expressing cortical interneurons J Neurosci 2010 30 15 5334 5345 10.1523/JNEUROSCI.5963-09.2010 20392955 PMC2919857

[B102] Zhao Y Flandin P Long JE Cuesta MD Westphal H Rubenstein JL Distinct molecular pathways for development of telencephalic interneuron subtypes revealed through analysis of Lhx6 mutants J Comp Neurol 2008 510 1 79 99 10.1002/cne.21772 18613121 PMC2547494

[B103] Fogarty M Grist M Gelman D Marin O Pachnis V Kessaris N Spatial genetic patterning of the embryonic neuroepithelium generates GABAergic interneuron diversity in the adult cortex J Neurosci 2007 27 41 10935 10946 10.1523/JNEUROSCI.1629-07.2007 17928435 PMC6672847

[B104] Xu Q Tam M Anderson SA Fate mapping Nkx2.1-lineage cells in the mouse telencephalon J Comp Neurol 2008 506 1 16 29 10.1002/cne.21529 17990269

[B105] Xu Q Wonders CP Anderson SA Sonic hedgehog maintains the identity of cortical interneuron progenitors in the ventral telencephalon Development 2005 132 22 4987 4998 10.1242/dev.02090 16221724

[B106] Xu Q Guo L Moore H Waclaw RR Campbell K Anderson SA Sonic hedgehog signaling confers ventral telencephalic progenitors with distinct cortical interneuron fates Neuron 2010 65 3 328 340 10.1016/j.neuron.2010.01.004 20159447 PMC2868511

[B107] Du T Xu Q Ocbina PJ Anderson SA NKX2.1 specifies cortical interneuron fate by activating Lhx6 Development 2008 135 8 1559 1567 10.1242/dev.015123 18339674

[B108] Flandin P Zhao Y Vogt D Jeong J Long J Potter G Westphal H Rubenstein JL Lhx6 and Lhx8 coordinately induce neuronal expression of Shh that controls the generation of interneuron progenitors Neuron 2011 70 5 939 950 10.1016/j.neuron.2011.04.020 21658586 PMC3153409

[B109] Zhao Y Marin O Hermesz E Powell A Flames N Palkovits M Rubenstein JL Westphal H The LIM-homeobox gene Lhx8 is required for the development of many cholinergic neurons in the mouse forebrain Proc Natl Acad Sci U S A 2003 100 15 9005 9010 10.1073/pnas.1537759100 12855770 PMC166428

[B110] Long JE Cobos I Potter GB Rubenstein JL Dlx1&2 and Mash1 transcription factors control MGE and CGE patterning and differentiation through parallel and overlapping pathways Cereb Cortex 2009 19 Suppl 1 i96 i106 19386638 10.1093/cercor/bhp045 PMC2693539

[B111] Long JE Swan C Liang WS Cobos I Potter GB Rubenstein JL Dlx1&2 and Mash1 transcription factors control striatal patterning and differentiation through parallel and overlapping pathways J Comp Neurol 2009 512 4 556 572 10.1002/cne.21854 19030180 PMC2761428

[B112] Kanatani S Yozu M Tabata H Nakajima K COUP-TFII is preferentially expressed in the caudal ganglionic eminence and is involved in the caudal migratory stream J Neurosci 2008 28 50 13582 13591 10.1523/JNEUROSCI.2132-08.2008 19074032 PMC6671763

[B113] Willi-Monnerat S Migliavacca E Surdez D Delorenzi M Luthi-Carter R Terskikh AV Comprehensive spatiotemporal transcriptomic analyses of the ganglionic eminences demonstrate the uniqueness of its caudal subdivision Mol Cell Neurosci 2008 37 4 845 856 10.1016/j.mcn.2008.01.009 18316204

[B114] Casarosa S Fode C Guillemot F Mash1 regulates neurogenesis in the ventral telencephalon Development 1999 126 3 525 534 9876181 10.1242/dev.126.3.525

[B115] Yun K Fischman S Johnson J Hrabe de Angelis M Weinmaster G Rubenstein JL Modulation of the notch signaling by Mash1 and Dlx1/2 regulates sequential specification and differentiation of progenitor cell types in the subcortical telencephalon Development 2002 129 21 5029 5040 12397111 10.1242/dev.129.21.5029

[B116] Horton S Meredith A Richardson JA Johnson JE Correct coordination of neuronal differentiation events in ventral forebrain requires the bHLH factor MASH1 Mol Cell Neurosci 1999 14 4–5 355 369 10588390 10.1006/mcne.1999.0791

[B117] Fode C Ma Q Casarosa S Ang SL Anderson DJ Guillemot F A role for neural determination genes in specifying the dorsoventral identity of telencephalic neurons Genes Dev 2000 14 1 67 80 10640277 PMC316337

[B118] Toresson H Potter SS Campbell K Genetic control of dorsal-ventral identity in the telencephalon: opposing roles for Pax6 and Gsh2 Development 2000 127 20 4361 4371 11003836 10.1242/dev.127.20.4361

[B119] Yun K Potter S Rubenstein JL Gsh2 and Pax6 play complementary roles in dorsoventral patterning of the mammalian telencephalon Development 2001 128 2 193 205 11124115 10.1242/dev.128.2.193

[B120] Waclaw RR Wang B Pei Z Ehrman LA Campbell K Distinct temporal requirements for the homeobox gene Gsx2 in specifying striatal and olfactory bulb neuronal fates Neuron 2009 63 4 451 465 10.1016/j.neuron.2009.07.015 19709628 PMC2772064

[B121] Panganiban G Rubenstein JL Developmental functions of the distal-less/Dlx homeobox genes Development 2002 129 19 4371 4386 12223397 10.1242/dev.129.19.4371

[B122] Eisenstat DD Liu JK Mione M Zhong W Yu G Anderson SA Ghattas I Puelles L Rubenstein JL DLX-1, DLX-2, and DLX-5 expression define distinct stages of basal forebrain differentiation J Comp Neurol 1999 414 2 217 237 10.1002/(SICI)1096-9861(19991115)414:2<217::AID-CNE6>3.0.CO;2-I 10516593

[B123] Liu JK Ghattas I Liu S Chen S Rubenstein JL Dlx genes encode DNA-binding proteins that are expressed in an overlapping and sequential pattern during basal ganglia differentiation Dev Dyn 1997 210 4 498 512 10.1002/(SICI)1097-0177(199712)210:4<498::AID-AJA12>3.0.CO;2-3 9415433

[B124] Anderson SA Eisenstat DD Shi L Rubenstein JL Interneuron migration from basal forebrain to neocortex: dependence on Dlx genes Science 1997 278 5337 474 476 10.1126/science.278.5337.474 9334308

[B125] Gelman DM Marin O Rubenstein JLR Noebels JL, Avoli M, Rogawski MA, Olsen RW, Delgado-Escueta AV The Generation of Cortical Interneurons Jasper’s Basic Mechanisms of the Epilepsies 2012 4 Bethesda (MD)

[B126] Gelman DM Martini FJ Nobrega-Pereira S Pierani A Kessaris N Marin O The embryonic preoptic area is a novel source of cortical GABAergic interneurons J Neurosci 2009 29 29 9380 9389 10.1523/JNEUROSCI.0604-09.2009 19625528 PMC6665570

[B127] Lodato S Rouaux C Quast KB Jantrachotechatchawan C Studer M Hensch TK Arlotta P Excitatory projection neuron subtypes control the distribution of local inhibitory interneurons in the cerebral cortex Neuron 2011 69 4 763 779 10.1016/j.neuron.2011.01.015 21338885 PMC3061282

[B128] Sugiyama S Di Nardo AA Aizawa S Matsuo I Volovitch M Prochiantz A Hensch TK Experience-dependent transfer of Otx2 homeoprotein into the visual cortex activates postnatal plasticity Cell 2008 134 3 508 520 10.1016/j.cell.2008.05.054 18692473

[B129] Beurdeley M Spatazza J Lee HH Sugiyama S Bernard C Di Nardo AA Hensch TK Prochiantz A Otx2 binding to perineuronal nets persistently regulates plasticity in the mature visual cortex J Neurosci 2012 32 27 9429 9437 10.1523/JNEUROSCI.0394-12.2012 22764251 PMC3419577

[B130] Petanjek Z Kostovic I Esclapez M Primate-specific origins and migration of cortical GABAergic neurons Front Neuroanat 2009 3 26 20011218 10.3389/neuro.05.026.2009 PMC2790953

[B131] Letinic K Zoncu R Rakic P Origin of GABAergic neurons in the human neocortex Nature 2002 417 6889 645 649 10.1038/nature00779 12050665

[B132] Yu X Zecevic N Dorsal radial glial cells have the potential to generate cortical interneurons in human but not in mouse brain J Neurosci 2011 31 7 2413 2420 10.1523/JNEUROSCI.5249-10.2011 21325508 PMC3079257

[B133] Fertuzinhos S Krsnik Z Kawasawa YI Rasin MR Kwan KY Chen JG Judas M Hayashi M Sestan N Selective depletion of molecularly defined cortical interneurons in human holoprosencephaly with severe striatal hypoplasia Cereb Cortex 2009 19 9 2196 2207 10.1093/cercor/bhp009 19234067 PMC2722430

[B134] Petanjek Z Berger B Esclapez M Origins of cortical GABAergic neurons in the cynomolgus monkey Cereb Cortex 2009 19 2 249 262 18477686 10.1093/cercor/bhn078 PMC2638783

[B135] Rakic S Zecevic N Early oligodendrocyte progenitor cells in the human fetal telencephalon Glia 2003 41 2 117 127 10.1002/glia.10140 12509802

[B136] Zecevic N Hu F Jakovcevski I Interneurons in the developing human neocortex Dev Neurobiol 2011 71 1 18 33 10.1002/dneu.20812 21154907 PMC3117059

[B137] Jakovcevski I Mayer N Zecevic N Multiple origins of human neocortical interneurons are supported by distinct expression of transcription factors Cereb Cortex 2011 21 8 1771 1782 10.1093/cercor/bhq245 21139075 PMC3138511

[B138] Tanaka DH Oiwa R Sasaki E Nakajima K Changes in cortical interneuron migration contribute to the evolution of the neocortex Proc Natl Acad Sci U S A 2011 108 19 8015 8020 10.1073/pnas.1102153108 21518872 PMC3093493

[B139] Corbin JG Nery S Fishell G Telencephalic cells take a tangent: non-radial migration in the mammalian forebrain Nat Neurosci 2001 4 Suppl 1177 1182 11687827 10.1038/nn749

[B140] Marin O Rubenstein JL A long, remarkable journey: tangential migration in the telencephalon Nat Rev Neurosci 2001 2 11 780 790 10.1038/35097509 11715055

[B141] Marin O Rubenstein JL Cell migration in the forebrain Annu Rev Neurosci 2003 26 441 483 10.1146/annurev.neuro.26.041002.131058 12626695

[B142] Metin C Baudoin JP Rakic S Parnavelas JG Cell and molecular mechanisms involved in the migration of cortical interneurons Eur J Neurosci 2006 23 4 894 900 10.1111/j.1460-9568.2006.04630.x 16519654

[B143] Lavdas AA Grigoriou M Pachnis V Parnavelas JG The medial ganglionic eminence gives rise to a population of early neurons in the developing cerebral cortex J Neurosci 1999 19 18 7881 7888 10479690 10.1523/JNEUROSCI.19-18-07881.1999 PMC6782477

[B144] Faux C Rakic S Andrews W Yanagawa Y Obata K Parnavelas JG Differential gene expression in migrating cortical interneurons during mouse forebrain development J Comp Neurol 2010 518 8 1232 1248 20151419 10.1002/cne.22271

[B145] Marsh ED Minarcik J Campbell K Brooks-Kayal AR Golden JA FACS-array gene expression analysis during early development of mouse telencephalic interneurons Dev Neurobiol 2008 68 4 434 445 10.1002/dneu.20602 18172891

[B146] Powell EM Mars WM Levitt P Hepatocyte growth factor/scatter factor is a motogen for interneurons migrating from the ventral to dorsal telencephalon Neuron 2001 30 1 79 89 10.1016/S0896-6273(01)00264-1 11343646

[B147] Powell EM Campbell DB Stanwood GD Davis C Noebels JL Levitt P Genetic disruption of cortical interneuron development causes region- and GABA cell type-specific deficits, epilepsy, and behavioral dysfunction J Neurosci 2003 23 2 622 631 12533622 10.1523/JNEUROSCI.23-02-00622.2003 PMC6741866

[B148] Friedman WJ Black IB Kaplan DR Distribution of the neurotrophins brain-derived neurotrophic factor, neurotrophin-3, and neurotrophin-4/5 in the postnatal rat brain: an immunocytochemical study Neuroscience 1998 84 1 101 114 10.1016/S0306-4522(97)00526-5 9522366

[B149] Fukumitsu H Furukawa Y Tsusaka M Kinukawa H Nitta A Nomoto H Mima T Furukawa S Simultaneous expression of brain-derived neurotrophic factor and neurotrophin-3 in Cajal-Retzius, subplate and ventricular progenitor cells during early development stages of the rat cerebral cortex Neuroscience 1998 84 1 115 127 10.1016/S0306-4522(97)00505-8 9522367

[B150] Maisonpierre PC Belluscio L Friedman B Alderson RF Wiegand SJ Furth ME Lindsay RM Yancopoulos GD NT-3, BDNF, and NGF in the developing rat nervous system: parallel as well as reciprocal patterns of expression Neuron 1990 5 4 501 509 10.1016/0896-6273(90)90089-X 1688327

[B151] Timmusk T Belluardo N Metsis M Persson H Widespread and developmentally regulated expression of neurotrophin-4 mRNA in rat brain and peripheral tissues Eur J Neurosci 1993 5 6 605 613 10.1111/j.1460-9568.1993.tb00526.x 8261135

[B152] Gorba T Wahle P Expression of TrkB and TrkC but not BDNF mRNA in neurochemically identified interneurons in rat visual cortex in vivo and in organotypic cultures Eur J Neurosci 1999 11 4 1179 1190 10.1046/j.1460-9568.1999.00551.x 10103114

[B153] Klein R Martin-Zanca D Barbacid M Parada LF Expression of the tyrosine kinase receptor gene trkB is confined to the murine embryonic and adult nervous system Development 1990 109 4 845 850 2171894 10.1242/dev.109.4.845

[B154] Brunstrom JE Gray-Swain MR Osborne PA Pearlman AL Neuronal heterotopias in the developing cerebral cortex produced by neurotrophin-4 Neuron 1997 18 3 505 517 10.1016/S0896-6273(00)81250-7 9115743

[B155] Fiumelli H Kiraly M Ambrus A Magistretti PJ Martin JL Opposite regulation of calbindin and calretinin expression by brain-derived neurotrophic factor in cortical neurons J Neurochem 2000 74 5 1870 1877 10800929 10.1046/j.1471-4159.2000.0741870.x

[B156] Arenas E Akerud P Wong V Boylan C Persson H Lindsay RM Altar CA Effects of BDNF and NT-4/5 on striatonigral neuropeptides or nigral GABA neurons in vivo Eur J Neurosci 1996 8 8 1707 1717 10.1111/j.1460-9568.1996.tb01314.x 8921261

[B157] Jones KR Farinas I Backus C Reichardt LF Targeted disruption of the BDNF gene perturbs brain and sensory neuron development but not motor neuron development Cell 1994 76 6 989 999 10.1016/0092-8674(94)90377-8 8137432 PMC2711896

[B158] Pozas E Ibanez CF GDNF and GFRalpha1 promote differentiation and tangential migration of cortical GABAergic neurons Neuron 2005 45 5 701 713 10.1016/j.neuron.2005.01.043 15748846

[B159] Airaksinen MS Saarma M The GDNF family: signalling, biological functions and therapeutic value Nat Rev Neurosci 2002 3 5 383 394 10.1038/nrn812 11988777

[B160] Canty AJ Dietze J Harvey M Enomoto H Milbrandt J Ibanez CF Regionalized loss of parvalbumin interneurons in the cerebral cortex of mice with deficits in GFRalpha1 signaling J Neurosci 2009 29 34 10695 10705 10.1523/JNEUROSCI.2658-09.2009 19710321 PMC6665705

[B161] Wichterle H Alvarez-Dolado M Erskine L Alvarez-Buylla A Permissive corridor and diffusible gradients direct medial ganglionic eminence cell migration to the neocortex Proc Natl Acad Sci U S A 2003 100 2 727 732 10.1073/pnas.242721899 12515855 PMC141064

[B162] Marin O Yaron A Bagri A Tessier-Lavigne M Rubenstein JL Sorting of striatal and cortical interneurons regulated by semaphorin-neuropilin interactions Science 2001 293 5531 872 875 10.1126/science.1061891 11486090

[B163] Tamamaki N Fujimori K Nojyo Y Kaneko T Takauji R Evidence that Sema3A and Sema3F regulate the migration of GABAergic neurons in the developing neocortex J Comp Neurol 2003 455 2 238 248 10.1002/cne.10476 12454988

[B164] Zimmer G Schanuel SM Burger S Weth F Steinecke A Bolz J Lent R Chondroitin sulfate acts in concert with semaphorin 3A to guide tangential migration of cortical interneurons in the ventral telencephalon Cereb Cortex 2010 20 10 2411 2422 10.1093/cercor/bhp309 20071458

[B165] Bagri A Marin O Plump AS Mak J Pleasure SJ Rubenstein JL Tessier-Lavigne M Slit proteins prevent midline crossing and determine the dorsoventral position of major axonal pathways in the mammalian forebrain Neuron 2002 33 2 233 248 10.1016/S0896-6273(02)00561-5 11804571

[B166] Marillat V Cases O Nguyen-Ba-Charvet KT Tessier-Lavigne M Sotelo C Chedotal A Spatiotemporal expression patterns of slit and robo genes in the rat brain J Comp Neurol 2002 442 2 130 155 10.1002/cne.10068 11754167

[B167] Whitford KL Marillat V Stein E Goodman CS Tessier-Lavigne M Chedotal A Ghosh A Regulation of cortical dendrite development by slit-robo interactions Neuron 2002 33 1 47 61 10.1016/S0896-6273(01)00566-9 11779479

[B168] Yuan W Zhou L Chen JH Wu JY Rao Y Ornitz DM The mouse SLIT family: secreted ligands for ROBO expressed in patterns that suggest a role in morphogenesis and axon guidance Dev Biol 1999 212 2 290 306 10.1006/dbio.1999.9371 10433822

[B169] Andrews W Barber M Hernadez-Miranda LR Xian J Rakic S Sundaresan V Rabbitts TH Pannell R Rabbitts P Thompson H The role of slit-robo signaling in the generation, migration and morphological differentiation of cortical interneurons Dev Biol 2008 313 2 648 658 10.1016/j.ydbio.2007.10.052 18054781

[B170] Andrews W Liapi A Plachez C Camurri L Zhang J Mori S Murakami F Parnavelas JG Sundaresan V Richards LJ Robo1 regulates the development of major axon tracts and interneuron migration in the forebrain Development 2006 133 11 2243 2252 10.1242/dev.02379 16690755

[B171] Andrews WD Barber M Parnavelas JG Slit-robo interactions during cortical development J Anat 2007 211 2 188 198 10.1111/j.1469-7580.2007.00750.x 17553100 PMC2375773

[B172] Barber M Di Meglio T Andrews WD Hernandez-Miranda LR Murakami F Chedotal A Parnavelas JG The role of Robo3 in the development of cortical interneurons Cereb Cortex 2009 19 Suppl 1 i22 i31 19366869 10.1093/cercor/bhp041 PMC2693537

[B173] Zimmer G Garcez P Rudolph J Niehage R Weth F Lent R Bolz J Ephrin-A5 acts as a repulsive cue for migrating cortical interneurons Eur J Neurosci 2008 28 1 62 73 10.1111/j.1460-9568.2008.06320.x 18662335

[B174] Rudolph J Zimmer G Steinecke A Barchmann S Bolz J Ephrins guide migrating cortical interneurons in the basal telencephalon Cell Adh Migr 2010 4 3 400 408 10.4161/cam.4.3.11640 20473036 PMC2958617

[B175] Sanchez-Alcaniz JA Haege S Mueller W Pla R Mackay F Schulz S Lopez-Bendito G Stumm R Marin O Cxcr7 controls neuronal migration by regulating chemokine responsiveness Neuron 2011 69 1 77 90 10.1016/j.neuron.2010.12.006 21220100

[B176] Tiveron MC Rossel M Moepps B Zhang YL Seidenfaden R Favor J Konig N Cremer H Molecular interaction between projection neuron precursors and invading interneurons via stromal-derived factor 1 (CXCL12)/CXCR4 signaling in the cortical subventricular zone/intermediate zone J Neurosci 2006 26 51 13273 13278 10.1523/JNEUROSCI.4162-06.2006 17182777 PMC6674999

[B177] Li G Adesnik H Li J Long J Nicoll RA Rubenstein JL Pleasure SJ Regional distribution of cortical interneurons and development of inhibitory tone are regulated by Cxcl12/Cxcr4 signaling J Neurosci 2008 28 5 1085 1098 10.1523/JNEUROSCI.4602-07.2008 18234887 PMC3072297

[B178] Flames N Long JE Garratt AN Fischer TM Gassmann M Birchmeier C Lai C Rubenstein JL Marin O Short- and long-range attraction of cortical GABAergic interneurons by neuregulin-1 Neuron 2004 44 2 251 261 10.1016/j.neuron.2004.09.028 15473965

[B179] Martini FJ Valiente M Lopez Bendito G Szabo G Moya F Valdeolmillos M Marin O Biased selection of leading process branches mediates chemotaxis during tangential neuronal migration Development 2009 136 1 41 50 10.1242/dev.025502 19060332

[B180] Yau HJ Wang HF Lai C Liu FC Neural development of the neuregulin receptor ErbB4 in the cerebral cortex and the hippocampus: preferential expression by interneurons tangentially migrating from the ganglionic eminences Cereb Cortex 2003 13 3 252 264 10.1093/cercor/13.3.252 12571115

[B181] Gong S Zheng C Doughty ML Losos K Didkovsky N Schambra UB Nowak NJ Joyner A Leblanc G Hatten ME A gene expression atlas of the central nervous system based on bacterial artificial chromosomes Nature 2003 425 6961 917 925 10.1038/nature02033 14586460

[B182] Alifragis P Liapi A Parnavelas JG Lhx6 regulates the migration of cortical interneurons from the ventral telencephalon but does not specify their GABA phenotype J Neurosci 2004 24 24 5643 5648 10.1523/JNEUROSCI.1245-04.2004 15201337 PMC6729337

[B183] Nobrega-Pereira S Kessaris N Du T Kimura S Anderson SA Marin O Postmitotic Nkx2-1 controls the migration of telencephalic interneurons by direct repression of guidance receptors Neuron 2008 59 5 733 745 10.1016/j.neuron.2008.07.024 18786357 PMC2643060

[B184] Cobos I Borello U Rubenstein JL Dlx transcription factors promote migration through repression of axon and dendrite growth Neuron 2007 54 6 873 888 10.1016/j.neuron.2007.05.024 17582329 PMC4921237

[B185] Cobos I Broccoli V Rubenstein JL The vertebrate ortholog of Aristaless is regulated by Dlx genes in the developing forebrain J Comp Neurol 2005 483 3 292 303 10.1002/cne.20405 15682394

[B186] Colombo E Collombat P Colasante G Bianchi M Long J Mansouri A Rubenstein JL Broccoli V Inactivation of Arx, the murine ortholog of the X-linked lissencephaly with ambiguous genitalia gene, leads to severe disorganization of the ventral telencephalon with impaired neuronal migration and differentiation J Neurosci 2007 27 17 4786 4798 10.1523/JNEUROSCI.0417-07.2007 17460091 PMC4916654

[B187] Kitamura K Yanazawa M Sugiyama N Miura H Iizuka-Kogo A Kusaka M Omichi K Suzuki R Kato-Fukui Y Kamiirisa K Mutation of ARX causes abnormal development of forebrain and testes in mice and X-linked lissencephaly with abnormal genitalia in humans Nat Genet 2002 32 3 359 369 10.1038/ng1009 12379852

[B188] Marsh E Fulp C Gomez E Nasrallah I Minarcik J Sudi J Christian SL Mancini G Labosky P Dobyns W Targeted loss of Arx results in a developmental epilepsy mouse model and recapitulates the human phenotype in heterozygous females Brain 2009 132 Pt 6 1563 1576 19439424 10.1093/brain/awp107 PMC2685924

[B189] Cuzon VC Yeh PW Cheng Q Yeh HH Ambient GABA promotes cortical entry of tangentially migrating cells derived from the medial ganglionic eminence Cereb Cortex 2006 16 10 1377 1388 16339085 10.1093/cercor/bhj084

[B190] Lopez-Bendito G Lujan R Shigemoto R Ganter P Paulsen O Molnar Z Blockade of GABA(B) receptors alters the tangential migration of cortical neurons Cereb Cortex 2003 13 9 932 942 10.1093/cercor/13.9.932 12902392

[B191] Cuzon Carlson VC Yeh HH GABAA receptor subunit profiles of tangentially migrating neurons derived from the medial ganglionic eminence Cereb Cortex 2011 21 8 1792 1802 10.1093/cercor/bhq247 21148088 PMC3202737

[B192] Crandall JE McCarthy DM Araki KY Sims JR Ren JQ Bhide PG Dopamine receptor activation modulates GABA neuron migration from the basal forebrain to the cerebral cortex J Neurosci 2007 27 14 3813 3822 10.1523/JNEUROSCI.5124-06.2007 17409246 PMC2711976

[B193] Ohtani N Goto T Waeber C Bhide PG Dopamine modulates cell cycle in the lateral ganglionic eminence J Neurosci 2003 23 7 2840 2850 12684471 10.1523/JNEUROSCI.23-07-02840.2003 PMC1201391

[B194] Sahara S Yanagawa Y O’Leary DD Stevens CF The fraction of cortical GABAergic neurons is constant from near the start of cortical neurogenesis to adulthood J Neurosci 2012 32 14 4755 4761 10.1523/JNEUROSCI.6412-11.2012 22492031 PMC3325497

[B195] Faux C Rakic S Andrews W Britto JM Neurons on the move: migration and lamination of cortical interneurons Neurosignals 2012 20 3 168 189 10.1159/000334489 22572780

[B196] Bortone D Polleux F KCC2 expression promotes the termination of cortical interneuron migration in a voltage-sensitive calcium-dependent manner Neuron 2009 62 1 53 71 10.1016/j.neuron.2009.01.034 19376067 PMC3314167

[B197] Elvevag B Goldberg TE Cognitive impairment in schizophrenia is the core of the disorder Crit Rev Neurobiol 2000 14 1 1 21 11253953

[B198] Sitskoorn MM Aleman A Ebisch SJ Appels MC Kahn RS Cognitive deficits in relatives of patients with schizophrenia: a meta-analysis Schizophr Res 2004 71 2–3 285 295 15474899 10.1016/j.schres.2004.03.007

[B199] Benes FM McSparren J Bird ED SanGiovanni JP Vincent SL Deficits in small interneurons in prefrontal and cingulate cortices of schizophrenic and schizoaffective patients Arch Gen Psychiatry 1991 48 11 996 1001 10.1001/archpsyc.1991.01810350036005 1747023

[B200] Akbarian S Kim JJ Potkin SG Hagman JO Tafazzoli A Bunney WE Jr Jones EG Gene expression for glutamic acid decarboxylase is reduced without loss of neurons in prefrontal cortex of schizophrenics Arch Gen Psychiatry 1995 52 4 258 266 10.1001/archpsyc.1995.03950160008002 7702443

[B201] Hashimoto T Volk DW Eggan SM Mirnics K Pierri JN Sun Z Sampson AR Lewis DA Gene expression deficits in a subclass of GABA neurons in the prefrontal cortex of subjects with schizophrenia J Neurosci 2003 23 15 6315 6326 12867516 10.1523/JNEUROSCI.23-15-06315.2003 PMC6740534

[B202] Lewis DA Sweet RA Schizophrenia from a neural circuitry perspective: advancing toward rational pharmacological therapies J Clin Invest 2009 119 4 706 716 10.1172/JCI37335 19339762 PMC2662560

[B203] Lisman JE Coyle JT Green RW Javitt DC Benes FM Heckers S Grace AA Circuit-based framework for understanding neurotransmitter and risk gene interactions in schizophrenia Trends Neurosci 2008 31 5 234 242 10.1016/j.tins.2008.02.005 18395805 PMC2680493

[B204] Barr MS Farzan F Tran LC Chen R Fitzgerald PB Daskalakis ZJ Evidence for excessive frontal evoked gamma oscillatory activity in schizophrenia during working memory Schizophr Res 2010 121 1–3 146 152 20598857 10.1016/j.schres.2010.05.023

[B205] Farzan F Barr MS Levinson AJ Chen R Wong W Fitzgerald PB Daskalakis ZJ Evidence for gamma inhibition deficits in the dorsolateral prefrontal cortex of patients with schizophrenia Brain 2010 133 Pt 5 1505 1514 20350936 10.1093/brain/awq046

[B206] Haenschel C Bittner RA Waltz J Haertling F Wibral M Singer W Linden DE Rodriguez E Cortical oscillatory activity is critical for working memory as revealed by deficits in early-onset schizophrenia J Neurosci 2009 29 30 9481 9489 10.1523/JNEUROSCI.1428-09.2009 19641111 PMC6666530

[B207] Uhlhaas PJ Singer W Abnormal neural oscillations and synchrony in schizophrenia Nat Rev Neurosci 2010 11 2 100 113 10.1038/nrn2774 20087360

[B208] Stefansson H Sarginson J Kong A Yates P Steinthorsdottir V Gudfinnsson E Gunnarsdottir S Walker N Petursson H Crombie C Association of neuregulin 1 with schizophrenia confirmed in a Scottish population Am J Hum Genet 2003 72 1 83 87 10.1086/345442 12478479 PMC420015

[B209] Harrison PJ Law AJ Neuregulin 1 and schizophrenia: genetics, gene expression, and neurobiology Biol Psychiatry 2006 60 2 132 140 10.1016/j.biopsych.2005.11.002 16442083

[B210] Wen L Lu YS Zhu XH Li XM Woo RS Chen YJ Yin DM Lai C Terry AV Jr Vazdarjanova A Neuregulin 1 regulates pyramidal neuron activity via ErbB4 in parvalbumin-positive interneurons Proc Natl Acad Sci U S A 2010 107 3 1211 1216 10.1073/pnas.0910302107 20080551 PMC2824309

[B211] Mei L Xiong WC Neuregulin 1 in neural development, synaptic plasticity and schizophrenia Nat Rev Neurosci 2008 9 6 437 452 18478032 10.1038/nrn2392 PMC2682371

[B212] Fazzari P Paternain AV Valiente M Pla R Lujan R Lloyd K Lerma J Marin O Rico B Control of cortical GABA circuitry development by Nrg1 and ErbB4 signalling Nature 2010 464 7293 1376 1380 10.1038/nature08928 20393464

[B213] Vullhorst D Neddens J Karavanova I Tricoire L Petralia RS McBain CJ Buonanno A Selective expression of ErbB4 in interneurons, but not pyramidal cells, of the rodent hippocampus J Neurosci 2009 29 39 12255 12264 10.1523/JNEUROSCI.2454-09.2009 19793984 PMC2774835

[B214] Millar JK Wilson-Annan JC Anderson S Christie S Taylor MS Semple CA Devon RS St Clair DM Muir WJ Blackwood DH Disruption of two novel genes by a translocation co-segregating with schizophrenia Hum Mol Genet 2000 9 9 1415 1423 10.1093/hmg/9.9.1415 10814723

[B215] Hikida T Jaaro-Peled H Seshadri S Oishi K Hookway C Kong S Wu D Xue R Andrade M Tankou S Dominant-negative DISC1 transgenic mice display schizophrenia-associated phenotypes detected by measures translatable to humans Proc Natl Acad Sci U S A 2007 104 36 14501 14506 10.1073/pnas.0704774104 17675407 PMC1964873

[B216] Niwa M Kamiya A Murai R Kubo K Gruber AJ Tomita K Lu L Tomisato S Jaaro-Peled H Seshadri S Knockdown of DISC1 by in utero gene transfer disturbs postnatal dopaminergic maturation in the frontal cortex and leads to adult behavioral deficits Neuron 2010 65 4 480 489 10.1016/j.neuron.2010.01.019 20188653 PMC3084528

[B217] Porteous DJ Millar JK Brandon NJ Sawa A DISC1 at 10: connecting psychiatric genetics and neuroscience Trends Mol Med 2011 17 12 699 706 10.1016/j.molmed.2011.09.002 22015021 PMC3253483

[B218] Ji Y Yang F Papaleo F Wang HX Gao WJ Weinberger DR Lu B Role of dysbindin in dopamine receptor trafficking and cortical GABA function Proc Natl Acad Sci U S A 2009 106 46 19593 19598 10.1073/pnas.0904289106 19887632 PMC2780743

[B219] Straub RE Jiang Y MacLean CJ Ma Y Webb BT Myakishev MV Harris-Kerr C Wormley B Sadek H Kadambi B Genetic variation in the 6p22.3 gene DTNBP1, the human ortholog of the mouse dysbindin gene, is associated with schizophrenia Am J Hum Genet 2002 71 2 337 348 10.1086/341750 12098102 PMC379166

[B220] Talbot K Eidem WL Tinsley CL Benson MA Thompson EW Smith RJ Hahn CG Siegel SJ Trojanowski JQ Gur RE Dysbindin-1 is reduced in intrinsic, glutamatergic terminals of the hippocampal formation in schizophrenia J Clin Invest 2004 113 9 1353 1363 15124027 10.1172/JCI20425 PMC398430

[B221] Weickert CS Straub RE McClintock BW Matsumoto M Hashimoto R Hyde TM Herman MM Weinberger DR Kleinman JE Human dysbindin (DTNBP1) gene expression in normal brain and in schizophrenic prefrontal cortex and midbrain Arch Gen Psychiatry 2004 61 6 544 555 10.1001/archpsyc.61.6.544 15184234

[B222] Ottis P Bader V Trossbach SV Kretzschmar H Michel M Leliveld SR Korth C Convergence of two independent mental disease genes on the protein level: recruitment of dysbindin to cell-invasive disrupted-in-schizophrenia 1 aggresomes Biol Psychiatry 2011 70 7 604 610 10.1016/j.biopsych.2011.03.027 21531389

